# From brown to white: Brown adipose tissue endothelial cells whiten in culture conditions

**DOI:** 10.1016/j.molmet.2026.102349

**Published:** 2026-03-12

**Authors:** Tabea Elschner, Stephan Grein, Jana Sander, Staffan Hildebrand, Lara Heubach, Nina Pannwitz, Maria Mircea, Elba Raimundez, Vasiliki Karagiannakou, Anastasia Georgiadi, Joerg Heeren, Jan Hasenauer, Alexander Pfeifer, Kerstin Wilhelm-Jüngling

**Affiliations:** 1 Institute for Cardiovascular Sciences, University Hospital, University of Bonn, Bonn, Germany; 2 Institute for Neurovascular Cell Biology, University Hospital, University of Bonn, Bonn, Germany; 3 Computational Biology, Life & Medical Sciences (LIMES) Institute, University of Bonn, Bonn, Germany; 4 Institute for Pharmacology and Toxicology, University Hospital, University of Bonn, Bonn, Germany; 5 Institute for Diabetes and Cancer, Helmholtz Diabetes Center, Helmholtz Centre Munich, German Research Center for Environmental Health, Neuherberg, Germany; 6 Department of Biochemistry and Molecular Cell Biology, University Medical Center Hamburg-Eppendorf, Hamburg, Germany

**Keywords:** Endothelial cells, brown adipose tissue, white adipose tissue, cell isolation, culture, heterogeneity, plasticity

## Abstract

Endothelial cells (ECs) are central regulators of vascular and metabolic homeostasis, yet their organ- and depot-specific diversity remains underexplored. Two major types of adipose tissue (AT) can be distinguished that differ substantially in their physiological function and vascularization: white AT (WAT), which is the major energy storage and brown AT (BAT), which is highly vascularized and dissipates energy [1–5]. While ECs from these depots likely contribute to adipose function, their characterization has been hindered by technical limitations in isolation and culture. Here, we establish a protocol for isolating and expanding ECs from murine BAT and WAT, enabling transcriptomic and functional analyses across depots. We demonstrate that freshly isolated BAT-ECs express depot-enriched gene signatures, including *Rgcc*, *Cdkn1c*, *Tcf15*, *Meox2*, and *Efnb1*, several of which are dynamically regulated during cold-induced BAT activation. These findings reveal novel BAT-EC markers and highlight specialized endothelial programs that may support BAT function. However, we also uncover that culturing BAT-ECs profoundly remodels EC identity. Transcriptomic profiling shows that BAT-ECs rapidly downregulate BAT-enriched endothelial markers and acquire features resembling WAT-ECs. This dedifferentiation is accompanied by signatures of proliferation, adhesion remodeling, and endothelial-to-mesenchymal transition. While these changes present challenges for maintaining depot-specific identity in culture, they also provide a framework to better interpret experimental outcomes and to investigate EC plasticity. Taken together, our study delivers a novel isolation and culture protocol for adipose ECs, defines BAT-EC markers, and demonstrates how culture conditions reshape their identity. These insights build the foundation for future research of AT vasculature.

## Highlights

• Fast method to purify endothelial cells (ECs) for direct RNA or protein extraction.
• Easy method to isolate and culture ECs specifically from thermogenic adipose tissue.
• Novel signature of BAT-ECs regulated upon browning and lost in culture.
• RGC-32, p57^kip2^ and EFNB1 as novel BAT-EC-enriched markers activated through β-adrenergic stimulation.

## 1 Introduction

Endothelial cells (ECs) line the inner surface of blood vessels and are essential regulators of tissue homeostasis, angiogenesis, and immune cell trafficking. Once thought to be relatively uniform, it is now well established that ECs exhibit a high degree of heterogeneity across organs and vascular beds [[1], [2], [3]]. Single-cell transcriptomic studies have demonstrated that ECs acquire organ-specific gene expression profiles in response to local cues such as shear stress, oxygen tension, growth factors, and interactions with surrounding parenchymal cells [1,4]. This endothelial diversity is not only structural but also functional, as ECs integrate tissue-specific signaling networks and actively participate in organ physiology and pathology [2].

*In vitro* culture of ECs has long been a valuable tool in vascular biology, providing a simplified and controllable system for studying endothelial function, angiogenic signaling, and vascular inflammation. Human umbilical vein endothelial cells (HUVECs), human dermal microvascular ECs (HDMECs), and immortalized lines such as EA.hy926 are among the most commonly used models. These cells have yielded key insights into EC biology and remain widely used due to their accessibility and ease of manipulation. However, it is increasingly clear that such generalized models may fail to recapitulate the unique characteristics of organ-specific ECs [3]. For example, ECs from the brain express tight junction proteins and transporters absent in other vascular beds [5], while liver sinusoidal ECs are fenestrated and display distinct scavenging functions [6]. Recently, also ECs of adipose tissues have been shown to vary between depots and to be highly influenced by the metabolic status of the organism [[7], [8], [9]]. The culture environment inevitably imposes adaptive changes on EC phenotype. When removed from their native microenvironment, ECs are forced to proliferate and therefore undergo a certain degree of remodeling. This includes the loss of *in vivo* cues such as laminar shear stress, extracellular matrix interactions, and intercellular communication, which together drive cells to adjust their transcriptome and growth behavior [10]. Rather than viewing this as a limitation, we propose that such remodeling reflects a necessary step for ECs to exit their highly specialized *in vivo* state and enable robust proliferation *in vitro*.

In metabolic research, the role of ECs is gaining increasing attention, especially adipose tissue (AT) EC. Importantly, not all adipose depots are the same: white adipose tissue (WAT) primarily stores energy, while brown adipose tissue (BAT) has thermogenic function and dissipates energy through mitochondrial uncoupling. In addition, to these major forms of AT, an intermediate form has been described, so called “beige fat” with the inguinal subcutaneous depot (WATi) containing the highest number of beige adipocytes, especially after cold exposure [[11], [12], [13], [14], [15]]. These differences are mirrored at the level of ECs. AT-ECs have been shown to regulate lipid transport, immune cell infiltration, and thermogenic programming [7,16,17]. Yet, despite their importance, depot-specific ECs remain under-characterized, largely due to challenges in their isolation and limited access to reliable culture systems. Therefore, most mechanistic insights are derived from non-depot-specific or immortalized cells. This raises critical questions: Can we develop a robust method to isolate and expand ECs from WAT and BAT? If so, do these cells exhibit a depot-specific identity *in vitro*, or do they converge toward a common, culture-induced phenotype?

In this study, we aimed to address these questions by establishing a robust protocol for the isolation and culture of adipose-derived ECs. Our method enables transcriptomic profiling and functional characterization of ECs from distinct adipose depots, while also uncovering the extent to which culture conditions influence their identity. Understanding how *in vitro* conditions shape adipose EC phenotypes is essential for interpreting data generated from culture models—and may also provide insight into how ECs respond to metabolic stress *in vivo*.

## 2 Experimental procedures

### 2.1 Animals

To establish the EC isolation protocol, mice of a C57BL6/J genetic background were used. For the isolation from pups, litters of early neonates were used, pooling 3–5 animals per isolation batch. For the isolation from adult mice, 8–16 week old mice were harvested and endothelial cells isolated from single mice. Both male and female mice were used. All animal experiments have been approved by the local authorities including the Committee for Animal Rights Protection of the State of North Rhine Westphalia (Landesamt für Natur, Umwelt und Verbraucherschutz: Az81–02.04.2022.A182).

### 2.2 Cold acclimation

C57BL6/J animals were kept in individually ventilated cages in a specific pathogen-free animal facility with controlled light–dark cycle (12 h–12 h), humidity (50–70%) and temperature (4–23 °C). Mice were housed individually with reduced cage bedding and allowed to acclimatise to the housing conditions for 48 h. Mice had access to food and water ad libitum throughout the experiments. For chronic cold exposure, this was followed by a decrease of housing temperatures to 16 °C for 72 h to allow for cold acclimatisation. Then, housing temperatures were decreased to 4 °C for seven days. Control animals were housed at 23 °C for twelve days. For acute cold exposure, the mice were injected subcutaneously with norepinephrine (1 mg/kg body weight, Arterenol®, CHEPLAPHARM) and allowed to rest for 48 h. Then, the mice were exposed to an acute cold stimulus of 4 °C for 1 h. The control animals were injected with an equal volume of PBS (Biowest, L0615-500), allowed to rest for 48 h and then harvested.

### 2.3 EC isolation

For EC isolation from pups, interscapular BAT and inguinal WAT of early neonates were dissected after decapitation. Adipose tissues of 3–5 neonates were pooled and processed one batch in the EC isolation protocol. Adult mice were killed by cervical dislocation and interscapular BAT, inguinal WAT and gonadal WAT were dissected. Tissues of adult mice were processed individually or pooled for up to three animals. For EC isolation after chronic cold exposure, adipose tissue of single mice was processed. Tissues were cut into small pieces and placed into adipose tissue dissociation mix in gentleMACS™ C tubes (Miltenyi Biotec, 130–0930237). For one batch of adipose tissue dissociation mix, 1.25 mL DMEM (Thermo Fisher Scientific, 11995-065) plus 50 μL Enzyme D, 25 μL Enzyme R and 6.25 μL Enzyme A (Miltenyi Biotec, 130-105-808) were used. Tissues were dissociated on gentleMACS™ Octo dissociator with heaters on “37C_mr_ATDK_1” setting. The cell suspension was filtered using 100 μm and 40 μm cell strainers, washing both the collection tubes and strainer with sterile 0.5% bovine serum albumin in PBS (PBS–B). The cell suspension was washed by centrifugation 300×*g* 5 min at room temperature and resuspension in 10 mL PBS-B. Through two consecutive centrifugation steps, the cell suspension was concentrated down to first 1 mL and then 90 μL cell suspension in PBS-B. Cells were incubated for 10 min on ice with FcR blocking reagent (Miltenyi Biotec, 130-092-575) to block unspecific binding.

The day before the intended EC isolation, Dynabeads™ (Thermo Fisher Scientific, 11035) were prepared for overnight coupling to CD31 antibody (BD Pharmigen, 553370). For this, 18 μL Dynabeads™ were washed with PBS-B on a magnetic separation rack (Cell Signaling Technology, 14654S) and resuspended in 296.25 μL PBS-B and 3.75 μL CD31 antibody in an 1.5 mL tube. The tube was mounted on an overhead rotator and rotated overnight at 4 °C. On the day of the experiment, the Dynabeads™ were washed in PBS-B to remove unbound antibody and resuspended in 300 μL antibody. After FcR blocking, the tissue samples were resuspended with 50 μL PBS-B and 50 μL washed antibody-coupled Dynabeads™. The sample tube was mounted on an overhead rotator and rotated for 1 h at room temperature. Using a magnetic separation rack, the bead-labelled cells were washed before resuspension in endothelial cell medium. Endothelial cell medium consisted of DMEM F12 (Thermo Fisher Scientific, 21041-025), 20% fetal calf serum (Thermo Fisher Scientific, 10270-160), 4 mL Endothelial Cell Growth Supplement/Heparin (ECGSH, PromoCell, C-30140), 1% Penicillin/Streptomycin (P 10,000 units/mL, S 10,000 μg/mL, Thermo Fisher Scientific, 15140122) and Amphotericin B (0.5 μg/mL, Carl Roth, 0246.1). For cultivation, the isolated ECs were seeded in 12 well plates at about 200,000 cells/well. The wells were pre-coated with 1% gelatin in H_2_O for 1 h at 37 °C 5% CO_2_ and then washed with 1× PBS. For neonate isolations, the protocol yielded on average 305,000 (±95,000) BAT-ECs and 470,000 (±260,000) WATi-ECs, for EC isolation from adult mice on average 120,000 (±90,000) BAT-ECs, 1,530,000 (±350,000) WATi-ECs and 1,430,000 (±470,000) WATg-ECs. After seeding, the cells were allowed to fully attach and the medium only changed the second day after isolation and cell seeding.

For RNA isolation, the isolated primary ECs were not resuspended in culture medium but snap-frozen in liquid nitrogen before undergoing the RNA isolation procedure.

### 2.4 Primary endothelial cell culture

The day after endothelial cell isolation, if ECs fully attached, the medium was gently changed to cell culture medium. If the cells did not fully attach yet, this procedure was postponed to the following day. Once grown to confluency on a 12-well, cells can be further passed onto one 6-well (P1), three 6-cm dishes (P2) and 10-cm dishes/T25 flasks (P3). For passaging, the medium was removed from the wells and they were gently washed with 1× PBS. Cells were then incubated with trypsin–EDTA 0.05% (Thermo Fisher Scientific, 25200056) in PBS at 37 °C 5% CO_2_ for 3–5 min. The wells were gently moved to detach the cells. If cells were still very attached, then can be further loosened using a cell scraper (SARSTEDT, 833950). The digestion reaction was stopped with the culture medium, using at least double the volume of trypsin–EDTA. The cell suspension was gently homogenized by pipetting and collected in 15 mL tubes. The cell suspension was centrifuged 300×*g* 5 min at room temperature and the supernatant removed. The cell pellet was then re-suspended in culture medium and the cells seeded on vessels that were pre-coated with 1% gelatin (Carl Roth, 0646.1) in H_2_O at 37 °C 5% CO_2_ and washed with 1× PBS. The cells were then further incubated at 37 °C 5% CO_2_. The cells can be kept up to passage P4, requiring medium changes every 2–3 days. For BAT-ECs, the cells can typically be reseeded every seven days and about every four days for WATi-ECs.

### 2.5 muMEC cell culture

CI-muMEC (InScreenEx, INS-Cl-1004) were cultured according to manufacturer's instructions on 2% gelatin-coated flasks in muMEC Medium (basal medium plus supplements, InScreenEx, INS-ME-1004) at 37 °C 5% CO_2_. Medium was changed every 2–3 days and cells were subcultivated at 70–90% confluence.

### 2.6 Immunohistochemistry

Endothelial cells were seeded onto glass bottom dishes (ibidi, 81218-200) that were coated with 1% gelatin (Carl Roth, 0646.1) in H_2_O at 37 °C 5% CO_2_ and washed with 1× PBS. Cells were allowed to reach 80–90% confluency before fixation. The cells were washed with 1× PBS, fixated with 4% paraformaldehyde (PFA, Sigma–Aldrich, P6148) for 15 min at room temperature. After fixation, the cells were washed thrice with 1× PBS and stored in PBS or stained directly. For staining, the cells were blocked by incubating with blocking buffer (3% BSA (Sigma–Aldrich, 1.12018.0100), 2% FCS (Gibco, 10270-160), 0.3% Tween®-20 (Sigma–Aldrich, SLCL7671) in PBS) for 1 h at room temperature.

For immunohistochemistry from whole tissue, interscapular BAT was dissected and fixated overnight at 4 °C in 10% formalin (G-Biosciences, 786–1058). Tissues were prepared for paraffin embedding using the automated embedding automate Microm STP-120 (Thermo Fisher Scientific). Samples were dehydrated in increasing isopropanol concentrations (70% for 3 h, two times 80% for 1 h each, two times 90% for 1 h each, 96% for 2 h and two times and 100% for 2 h each) followed by two steps in xylene (two times 1 h each) to clear out the isopropanol. Samples were then incubated in molten paraffin grade 3 wax (two times 1 h each). The paraffin-infiltrated samples were embedded and casted into molds with liquid paraffin using the Microm EC 350 (Thermo Fisher Scientific) tissue embedding center. The tissues were sectioned into 7 μm slices using a Microm HM355 S microtome (Thermo Fisher Scientific) and mounted on Superfrost™ Plus Adhesion microscope slides (New Erie Scientific LLC). The paraffin-embedded sections were deparaffinized by incubation in xylene (three times, 15 min each) and a 1:1 ratio of xylene and ethanol for 5 min. The samples were rehydrated in a graded ethanol series (three times 100% 2 min each, two times 90% 2 min each, two times 70 % 2 min each) followed by two water rinses for 2 min each. The samples underwent target antigen retrieval using citrate buffer (Sigma–Aldrich, C9999) for 10 min at 95 °C in a water bath and allowed to cool down to room temperature (30–45 min). Slides were then washed with 1× PBS (twice for 2 min each, once for 10 min) and rinsed twice for 10 min (rinsing solution 0.2% gelatin (Sigma–Aldrich, G7041) plus 0.25% Triton X-100 (Sigma–Aldrich, T8787) in 0.5× PBS in H_2_O). Sample were encircled with hydrophobic pen and blocked in blocking buffer (3% BSA, 2% FCS, 0.3% Tween®-20, 0.25% Triton X-100 in PBS) overnight in a humidified chamber at 4 °C. Subsequent staining steps for tissue sections were also performed in a humidified chamber.

Primary antibodies were diluted in blocking buffer with surface markers at 1:200 and ERG (Abcam, ab92513) at 1:500 dilution. Primary antibodies were incubated in blocking buffer at 4 °C overnight (CD31 BD Pharmigen, 553370; VE-Cadherin R&D Systems, AF1002). Following washes with PBS, samples were incubated with Alexa-conjugated secondary antibodies at 1:500 dilution (TOPRO 1:2000 (Thermo Fischer Scientific, T3605)) for 2 h at 4 °C. After washes with PBS, tissue sections were stained with DAPI (1:1000 in PBS (Carl Roth, 6843.1)) 10 min at 4 °C. All samples were mounted using Fluoromount G (SouthernBiotech, 0100-01).

Representative images were acquired using a Leica SP8 confocal microscope (Leica Microsystems GmbH). For image processing, Fiji/ImageJ and Illustrator (Adobe Inc.) software were used.

### 2.7 Immunoblotting

For protein isolation, samples were lysed in RIPA buffer (Sigma–Aldrich, #R0278; 150 mM NaCl, 1.0% (v/v) IGEPAL CA-630, 0.5% sodium deoxycholate, 0.1% SDS, and 50 mM Tris, pH 8.0) supplemented with 1× EDTA-Free Complete Protease Inhibitor Cocktail (Roche, 11836170001), PhosSTOP™, Phosphatase Inhibitor cocktail mix (Roche, 4906845001) and 1 mM phenylmethylsulfonyl fluoride (Sigma–Aldrich, 10837091001).

Cultured ECs and muMECs were grown to about 90% confluency, washed with PBS and snap-frozen on dry ice. For protein isolation, 150 μL lysis buffer were added and the cells detached and disintegrated by scraping down and transferred into a 1.5 mL tube.

For protein analysis from tissue, interscapular BAT, inguinal WAT or gonadal WAT tissue pieces were snap-frozen in liquid nitrogen directly after the harvest and stored at −80 °C. Samples were manually mashed using Rotilabor®-micro pestle (Carl Roth, YE14.1) and the tissue integrity further disrupted by re-freezing on dry ice and grinding the tissue at least three times. Depending on the tissue type, the tissue mash was resuspended in different amounts of supplemented RIPA buffer (BAT and WATi: 600 μL, WATg: 300–400 μL buffer).

Protein lysates were stored on ice for 30 min and the samples were centrifuged for 15 min 17000×*g* at 4 °C. The supernatant was transferred into a new 1.5 mL tube and samples stored at −80 °C. For tissue protein samples, the centrifugation and supernatant transfer step was performed twice. The protein concentration was determined using a BSA protein standard (Sigma–Aldrich, P0834-10X1ML) and ROTI Quant Bradford solution (Carl Roth, K015.1). The protein concentration at 595 nM was measured using a Spark® Multimode microplate reader (Tecan).

Protein samples were diluted in 4× Laemmli buffer (Thermo Fisher Scientific, J60015), boiled at 95 °C for 10 min and stored at −20 °C. The proteins were separated by SDS-PAGE (Tris-glycine gels with Tris/glycine/SDS buffer, Bio-Rad, 1610772) using 12% acrylamide gels (TGX FastCast Acrylamide Kit 12%, Bio-Rad, 1610175) according to manufacturers’ instructions. Samples and Precision Plus Protein™ Standards Dual Color (Bio-Rad, 161–0374) were loaded on 12% acrylamide gels and separated in an electric field of 90 V for varying time spans. Proteins were transferred onto nitrocellulose membranes (Trans-blot turbo RTA transfer kit, Bio-Rad, 1704271) using the Trans-Blot® Turbo™ Transfer System (Bio-Rad). Membranes were stained with Ponceau S solution (0.1% Ponceau, 5% acetic acid) to evaluate equal gel loading and transfer. Membranes were rinsed twice in TBS-T (0.01% Tween-20 in TBS) and blocked for 1 h in 5% milk in TBS-T. Membranes were incubated with primary antibodies at a 1:1000 dilution in 5% milk in TBS-T or 5% BSA in TBS-T overnight at 4 °C. After washes and a consecutive 30 min 5% milk block, horseradish peroxidase-coupled secondary antibodies at a 1:5000 dilution were incubated for 1.5 h at room temperature rocking at 20 U/min. To detect chemiluminescent protein signal, membranes were incubated in an ECL detection kit (Clarity™ Western ECL Substrate, Bio-Rad, 170–5061) and imaged using ChemiDoc™ MP Imaging System (Bio-Rad). Band intensities were quantified using the Image Lab software (Bio-Rad) and visualized using Photoshop (Adobe Inc.) and Illustrator (Adobe Inc.).

### 2.8 Gene expression analysis

RNA isolation from primary ECs snap-frozen after the isolation was performed using RNeasy® Plus Micro Kit (Quiagen, 74034). RNA Isolation Kit was used according to the manufacturer's instructions, incuding β-mercaptoethanol addition in the lysis step and eluting in 20 μL H_2_O. For primary ECs, cDNA synthesis was performed using High Capacity cDNA Reverse Transcription Kit (Thermo Fisher Scientific, 4368814).

RNA isolation from cultured ECs and muMECs was performed using the NucleoSpin® RNA Plus Isolation Kit (Machery-Nagel, 74084.250). Cells were grown to about 90% confluency, washed with PBS and snap-frozen on dry ice. For RNA isolation, 350 μL of lysis buffer were added and the cells detached and disintegrated by scraping down. The cell lysate was collected in a 1.5 mL tube and the RNA Isolation Kit was used according to the manufacturer's instructions, with one modification. During the elution step, the volume of elution buffer was reduced to 20 μL, which was subsequently reused for a second elution by adding it again to the RNA-binding column. RNA concentration was determined using a NanoDrop One (Thermo Fisher Scientific), typically yielding concentrations between 50 and 100 ng/μL. For cDNA synthesis, 500 ng of RNA was used. cDNA synthesis was performed using the LunaScript RT Supermix Kit (New England Biolabs, E3010G).

For quantitative PCR (qPCR), TaqMan probes were employed to detect the target genes of interest. Beta-actin was selected as the reference gene due to its stable expression across various tissues and experimental conditions. The relative expression of the target genes was calculated using the ΔΔCt method, where the Ct value of the target gene was normalized to the Ct value of beta-actin to account for variations in RNA input or reverse transcription efficiency. Ct values were measured using QuantStudio 3 (Thermo Fisher Scientific).

### 2.9 Bulk RNA sequencing

For RNA-Seq, RNA was isolated from primary adult ECs from BAT, WATi and WATg depots as well as neonate primary and cultured (passage P1) BAT-ECs using the RNeasy® Plus Micro Kit (Quiagen, 74034). RNA Isolation Kit was used according to the manufacturer's instructions, incuding β-mercaptoethanol (Sigma–Aldrich, M3148) addition in the lysis step. Bulk RNA sequencing way performed with Recipient Biomarker Technologies (BMK) GmbH. Briefly, RNA sample purity, concentration and integrity as well as library preparation quality were analysed by NanoDrop, Qubit 2.0 and Agilent 2100. The qualified library was sequenced using eukaryotic mRNA sequencing with a PE150 mode. Clean data of high quality was retrieved by filtering Raw data, thereby removing adapter sequence and low-quality reads. The obtained clean data was mapped to *Mus musculus* reference genome (Ensembl, Version GRCm38_release79) to generate mapped data. As library quality control, insert length and sequencing randomness were assessed on mapped data. RNA sequencing data set was analysed for gene expression quantification and principal component analyses (PCA) were performed on FPKM of each sample with similarity among them illustrated by reducing dimensionality into two or three principal components. Gene expression from adult BAT-ECs and WAT-ECs as well as neonate primary and cultured BAT-ECs was compared and differentially expressed genes (DEG) were determined using DESeq2 [18]. DEG were characterized by Fold Changes (FC) ≥2 and p-values <0.05. Gene Set Enrichment Analysis (GSEA) was performed.

#### 2.9.1 Dendrogram

Two bulk RNA-seq datasets, containing primary BATECs and primary WATgECs, respectively cultivated BATECs and isolated BATECs have been merged into a consolidated dataset with a total of 4 cell types.

Prior to cluster analysis, genes with low expression were filtered by retaining only those with a raw count of at least 10 in a minimum of two samples. Variance-stabilized expression values of the remaining genes were calculated and considered for hierarchical clustering. Pairwise sample similarities were quantified using the Pearson correlation coefficient (ρ) and transformed into a dissimilarity measure defined by 1 – ρ.

Agglomerative hierarchical clustering was performed using the unweighted pair group method with arithmetic mean (UPGMA) on the adipose tissue hallmark genes [19]. Resulting dendrogram leafs (samples) were color coded by one of the four corresponding cell types. Visualized are the correlation-based relationships between samples, with branch lengths representing expression dissimilarity.

### 2.10 Single-cell RNA-sequencing reanalysis

#### 2.10.1 Data description

A published scRNA-seq dataset of cells isolated from the stromal vascular fraction of BAT (BAT-SVF) from mice housed at either thermoneutral (TN: 30 °C for 1 week), room temperature (RT: 22 °C) or cold (5 °C for 2 days or 7 days) was downloaded from NCBI GEO database under accession number GSE160585, subsequently preprocessed and analyzed with Seurat v5 as detailed below.

#### 2.10.2 Preprocessing

Raw count matrices from GSE160585 were imported using the Read10× function in Seurat. Metadata provided with the dataset, as well as cluster identity information from supplementary tables, was incorporated into the Seurat object. For each sample, normalization was performed with SCTransform while regressing out cell cycle differences. The datasets were then integrated by selecting 3,000 features, computing anchors, and applying Seurat's SCT-based integration workflow. Principal component analysis (PCA) was performed, followed by dimensionality reduction with UMAP based on the first 50 principal components. The number of components was determined by considering an inspection of the elbow plot, automated detection and expert review. A shared nearest–neighbor graph was constructed, and clustering was carried out at multiple resolutions (0.1–1.8).

Cluster annotation was guided by known marker genes, supplementary information from the original publication, and the identification of cluster-specific markers using FindAllMarkers. Cell identities were reassigned accordingly (e.g., endothelial cell subtypes, vascular smooth muscle cells, adipocytes, Schwann cells, pericytes, adipocyte progenitors, and immune cell subsets such as B cells, T cells, NK cells, monocytes, and macrophages). Custom color palettes were defined for visualization in UMAP plots. To focus on stromal and vascular populations, immune cells were collapsed into a single category and subsequently excluded, generating a subset object devoid of immune cells. The processed objects were saved as .rds files for downstream analyses.

#### 2.10.3 DE-analysis

Analysis was conducted with the pre-integrated Seurat object provided as an .rds file from the preprocessing steps, which had already undergone quality control, normalization, and integration using the standard workflow in Seurat. We applied 50 dimensions throughout all analysis to ensure consistency across visualizations. Condition-specific embeddings were generated with the DimPlot function, and a custom color palette was applied to distinguish temperature treatments. For gene-level exploration, expression of set of genes was projected onto the UMAP embeddings using FeaturePlot, stratified by sample condition (temperature), and arranged into two-column grids with wrap_plots to enable direct comparison of expression patterns across conditions. Additionally lists for differentially expressed genes was created for pairwise comparisons of control and perturbations and provided as Excel files reporting p-value and log2FC of normalized RNA expression values from the corresponding assay, accompanying condition-specific Violin plots have been generated. DE testing used the default Wilcoxon rank-sum test with Bonferroni correction. Cell identities were confirmed with marker genes. Violin plots of gene expression has been reported for different gene groups in addition.

The preprocessing and analysis scripts have been deposited the following Github repository: https://github.com/stephanmg/shamsi-BAT-re-analysis.

### 2.11 Quantification and statistical analysis

Statistical analysis was carried out using PRISM 10 (GraphPad Software) on the raw data. Comparisons between two groups were made using a t-test, unless otherwise specified in the figure legends. A p-value of p < 0.05 was considered statistically significant. Data are presented as dot plots, with vertical bars representing the means, unless otherwise noted in the figure legends.

### 2.12 Reanalysis of human single-cell RNASeq data

Publicly available single-cell RNA-sequencing datasets were obtained from the Expression Atlas (accession codes E-MTAB-8564 and E-MTAB-9199), comprising brown and white adipose tissue (BAT and WAT). Both datasets were processed using the standard Seurat workflow, including dimensionality reduction and UMAP-based visualization of cell types. Gene expression patterns were explored using FeaturePlots, ViolinPlots, and UMAP embeddings for selected cell populations following differential expression analysis.

## 3 Results

### 3.1 Isolation of endothelial cells (ECs) from neonatal and adult adipose tissue

**Figure 1:**
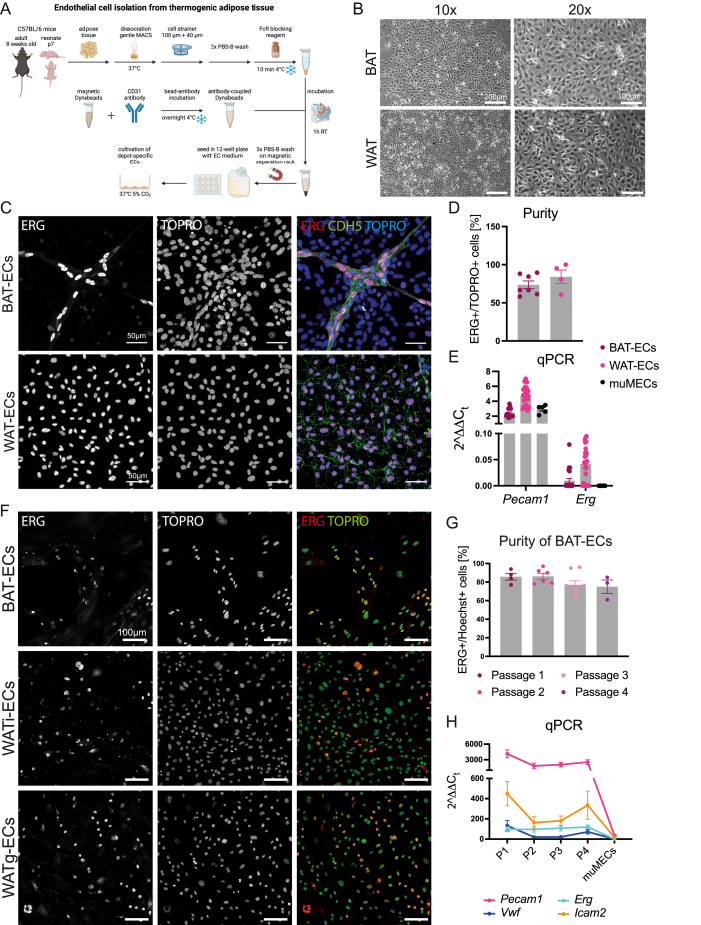
**Our endothelial isolation protocol gives rise to high purity of adipose ECs A** Schematic representation of the major steps of endothelial cell isolation from thermogenic adipose tissue. **B** Brightfield images of endothelial cells isolated from brown adipose tissue (top panels) and white adipose tissue (bottom panels) at different magnifications (10×, 20×). **C** Confocal images of BAT-ECs (top) and WAT-ECs (bottom) isolated from neonatal mice and labelled for ERG, TOPRO and CDH5 (VE-Cadherin). **D** Purity of neonatal endothelial cultures (passage 1) of BAT (n = 7 independent samples) and WAT (n = 4 independent samples) determined from culture dishes labelled for ERG and TOPRO. **E** mRNA levels of the endothelial markers *Pecam1* and *Erg* at early passages of BAT-ECs (n = 23 independent samples) and WAT-ECs (n = 28 independent samples) in comparison to the immortalized murine EC line, muMECs (n = 5 independent samples). This displays high endothelial marker expression of BAT-ECs and WAT-ECs. **F** Representative confocal images of BAT-ECs, WATi-ECs and WATg-ECs isolated from adult mice and stained for ERG and TOPRO, displaying high purity of the endothelial cultures. **G** Quantification of BAT-EC purity at different passages (n ≥ 3 independent samples), showing that purity only mildly drops from one passage to the other. **H** qPCR results of endothelial markers (*Pecam1, Erg, Vwf, Icam2*) of BAT-ECs at different passages (n ≥ 3 independent samples), representing high levels of endothelial markers at different passages in comparison to muMECs. (For interpretation of the references to color in this figure legend, the reader is referred to the Web version of this article.)

This protocol was developed to isolate ECs from interscapular brown adipose tissue (BAT), inguinal white adipose tissue (WATi) and gonadal white adipose tissue (WATg). A step-by-step procedure of the isolation process is presented in Figure 1A.

In brief, AT was initially dissociated by chopping and enzymatic digestion. The resulting cell suspension was then filtered through 100 μm and 40 μm cell strainers to obtain a single-cell suspension. After washing, nonspecific binding was blocked. Subsequently, the single-cell suspension was incubated with CD31-coupled magnetic beads. Using magnetic separation racks, the bead-labeled cells were washed and then resuspended in EC culture medium (DMEM-F12 supplemented with 20% FCS, endothelial growth factors, and antibiotics).

The isolated ECs were seeded onto gelatin-coated 12-well plates at a density of approximately 200,000 cells per well. For the isolation of ECs from adult fat depots, the tissues do not need to be pooled, allowing for the comparison of ECs from mice of different genotypes. In contrast, for the isolation of ECs from neonatal adipose tissue, fat depots from 3 to 5 neonates were pooled to ensure a sufficient number of ECs for culture. For neonatal isolations, the protocol yielded an average of 305,000 (±95,000) BAT-ECs and 470,000 (±260,000) WATi-ECs, when fat depots of four pups were pooled. In contrast, EC isolations from adult mice resulted in an average yield of 120,000 (±90,000) BAT-ECs, 1,530,000 (±350,000) WATi-ECs and 1,430,000 (±470,000) WATg-ECs per single fat depot. After seeding, the cells were allowed to fully attach for two days before the culture medium was changed. At this stage, the cells already exhibited the characteristic cobblestone morphology of ECs (Supplementary Fig. 1A).

Once the cells reached confluency in a 12-well plate, they were passaged to a 6-well plate (passage 1). Upon reaching confluency again, the cells were split into three 6-cm dishes (passage 2). In the third passage, cells from one confluent 6-cm dish were seeded onto a 10-cm dish or T25 flask. The cells could be maintained up to passage P4, with medium changes required every 2–3 days. BAT-ECs could be reseeded approximately every seven days, whereas WAT-ECs reached confluency in about four days.

### 3.2 The protocol gives rise to a high purity of ECs

A fast and cost-effective quality control method for EC cultures is the assessment of cell morphology. ECs are typically characterized by a cobblestone-like shape, which serves as a criterion for high-quality EC cultures. For the successful culture of ECs, it is essential to minimize contamination by mesenchymal cells, particularly fibroblasts. Fibroblasts exhibit a lower doubling rate than ECs and can rapidly overgrow EC cultures within a single passage (Supplementary Fig. 1B). In contrast to ECs, fibroblasts look spikier and do not grow in monolayers. The purified BAT- and WAT-ECs isolated using our protocol display a uniform cobblestone monolayer (Figure 1B), suggesting a high degree of endothelial purity.

While flow cytometry is commonly used to assess EC purity in other protocols [20,21], this approach was not suitable in our case. The isolated cells retained large surface-bound Dynabeads that substantially distorted scatter profiles and produced unstable flow signals on our cytometer. Thus, flow-cytometric purity assessment was not technically reliable in our setup. Instead, we implemented an alternative method to evaluate and quantify cell purity. We seeded approximately 40,000 isolated cells from the first passage onto small staining dishes and subsequently performed immunofluorescent staining. ECs were identified using an antibody specific for the ETS transcription factor ERG, which labels the cell nucleus [22]. By co-staining with a nuclear marker, such as TOPRO, we were able to distinguish and quantify double-positive cells as ECs (Figure 1C). Using this approach, our isolation protocol consistently achieved a purity of approximately 75% for brown adipose tissue-derived ECs (BAT-ECs) and 85% for ECs isolated from inguinal white adipose tissue (WAT-ECs) in the first passage (Figure 1D).

In addition to immunofluorescence-based purity assessment, we isolated RNA from the cultured cells to examine the expression of key blood endothelial markers, including *Pecam1* and *Erg.* We compared these primary ECs to early-passage commercially available, immortalized murine capillary ECs isolated from the mouse lung (muMECs, InScreenEx) (Figure 1E): BAT-ECs exhibited slightly lower expression of *Pecam1*, but clearly higher *Erg*, while WAT-ECs showed more abundant transcript levels for both. Additionally, BAT- and WAT-ECs displayed higher endothelial marker expression compared to muMECs, further underlining the endothelial identity of BAT- and WAT-ECs (Supplementary Fig. 1C).

In summary, the EC isolation protocol described here, yields highly pure endothelial cells. Further, the findings confirm that our protocol not only enables the isolation of ECs from AT but also maintains endothelial characteristics under our culture conditions, providing an additional quality criterion for this method.

### 3.3 The isolation protocol is effective in adult mice

Consistent with the results obtained from neonatal fat depot isolations, the protocol achieves high EC purity in adult tissue-derived ECs. Staining for the endothelial-specific nuclear marker ERG and comparing it to total nuclear staining with TOPRO or Hoechst revealed a BAT-EC purity of approximately 82%, which declined slightly from passage 1 to passage 4 (Figure 1F,G).

To further validate the endothelial identity of the isolated cells, we quantified transcript levels of canonical EC markers (*Pecam1*, *Erg*, *Icam2*, *Vwf*) in BAT-ECs and compared them to those in muMECs. Further, we followed the marker expression over four passages (Figure 1H). All canonical endothelial markers were highly expressed in primary BAT-ECs, whereas their expression levels in muMECs were significantly lower or barely detectable.

These findings confirm the robustness and reliability of our protocol for isolating ECs from adult adipose tissue and further emphasize the advantages of using primary ECs over immortalized endothelial cell lines for physiologically relevant studies.

### 3.4 ECs isolated from BAT and WATg express depot-specific signatures

**Figure 2:**
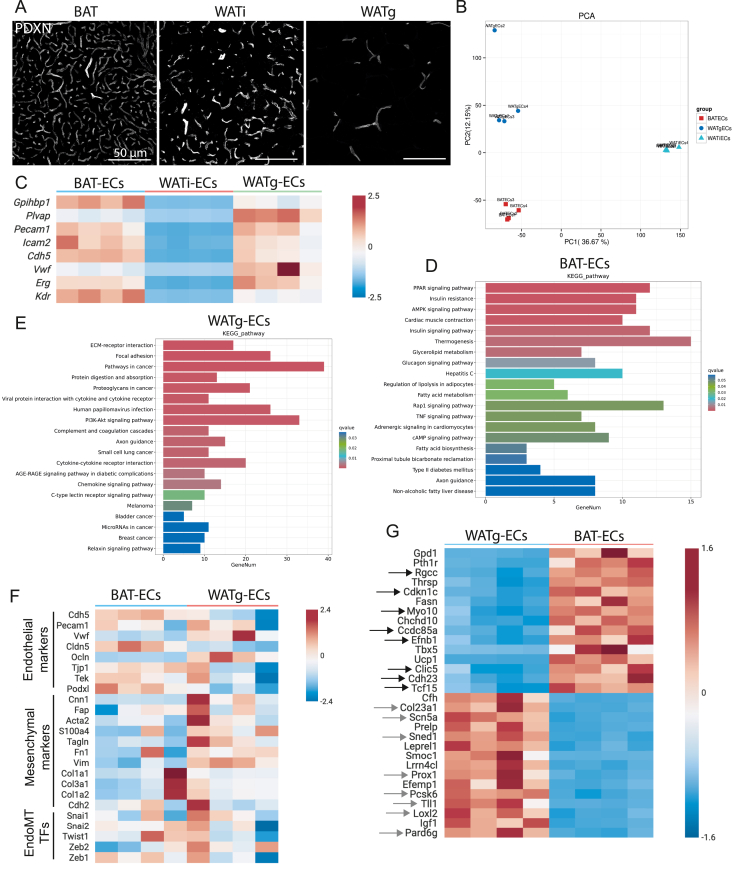
**Transcriptomic analysis of isolated BAT-ECs defines novel BAT-enriched EC markers A** Podocalyxin (PDXN) immunofluorescence labeled BAT (left), WATi (middle) and WATg (right) of C57Bl6 mice housed at 23 °C, presenting different densities in their vascular network. **B** Principal Component Analysis (PCA) comparing the endothelial transcriptome of 4 replicates of each fat depot (BATECs, WATiECs, WATgECs) representing low variability on y-axis (PC2) but high deviation of WATiECs on x-axis (PC1). **C** Heatmap of endothelial marker gene expression measured through mRNA sequencing of BAT-ECs, WATi-ECs and WATg-ECs, showing that WATi-ECs express endothelial markers only at very low levels. BAT-EC marker *Gpihbp1* is highly abundant in BAT-ECs, while WAT-EC endothelial marker *Plvap* is strongly transcribed in WATg-ECs. **D** KEGG pathway analysis using all differentially upregulated genes in BAT-ECs compared to WATg-ECs. **E** KEGG pathway analysis of significantly upregulated gene in WATg-ECs compared to BAT-ECs. **F** Heatmap of genes involved in endothelial-to-mesenchymal transition (EndoMT) revealing that endothelial markers are lower in WATg-ECs, while mesenchymal marker and EndoMT transcription factors are more abundant in WATg-ECs compared to BAT-ECs. **G** Heatmap of the 15 most significantly enriched genes in BAT-ECs compared to WATg-ECs and the 15 most significantly upregulated genes in WATg-ECs compared to BAT-ECs.

As noted in the introduction, adipose tissue exists in distinct types - brown, white, and beige - each with characteristic functions in energy dissipation and storage. In addition to these metabolic roles, adipose depots differ in their degree of vascularization (Figure 2A): BAT is densely vascularized, WATg is sparsely vascularized, and WATi exhibits intermediate vascular density. Given these differences, we sought to determine whether BAT-ECs exhibit a distinct transcriptomic profile compared with ECs from white depots, and to identify the signaling pathways that distinguish them. To this end we assessed the transcriptomes of ECs freshly isolated from BAT, WATi and WATg using our protocol. RNA was extracted immediately from the primary cells and subjected to RNA sequencing. Principal component analysis (PCA) showed that overall gene expression was highly similar among ECs isolated from the same fat depot (Figure 2B). Along principal component 2 (PC2), WATi-ECs, originating from a beige-fat depot, clustered between BAT-ECs, representing pure brown fat, and WATg-ECs, representing the whitest depot. Along PC1, however, WATi-ECs clustered far from both BAT- and WATg-ECs. When examining canonical endothelial markers, WATi-ECs displayed very low expression levels, and were therefore excluded from further analysis (Figure 2C). By contrast, BAT-ECs and WATg-ECs expressed high levels of canonical endothelial markers along with depot-enriched markers *Gpihbp1* and *Plvap*, respectively (Figure 2C). Subsequent comparative analysis focused on BAT-ECs versus WATg-ECs. KEGG pathway analysis confirmed the specificity of our approach, with significantly enriched pathways related to adipocyte metabolism, including PPAR signaling, insulin signaling, thermogenesis, fatty acid metabolism, and regulation of lipolysis (Figure 2D, Supplementary Table 1). These results suggest that BAT-ECs may directly support adipocyte metabolic function.

In contrast, KEGG pathway analysis of genes enriched in WAT-ECs highlighted pathways related to extracellular matrix–receptor interactions, focal adhesion, cancer-associated signaling, and PI3K–Akt signaling (Figure 2E, Supplementary Table 1). Closer inspection of the genes contributing to these pathways revealed that many are known to be associated with endothelial-to-mesenchymal transition (EndoMT). Consistent with this, the heatmap in Figure 2F shows that, compared with BAT-ECs, WAT-ECs display reduced expression of canonical endothelial markers alongside increased expression of mesenchymal-associated genes. Together, these findings suggest that WAT-ECs adopt a transcriptional state that is more closely aligned with EndoMT-related programs relative to BAT-ECs.

To further characterize endothelial features predominantly associated with specific adipose depots, we first explored the 15 most highly enriched genes in BAT-ECs and WAT-ECs (Figure 2G). To confirm that the 15 identified BAT-EC genes are particularly enriched in ECs within murine BAT, we compared them with published single-cell RNA-seq data from murine BAT [23]. This approach allowed us to validate that the selected markers reflect endothelial enrichment within the tissue context. The analysis revealed that eight of the top 15 genes were either enriched (*Rgcc, Tcf15*) or exceptionally expressed (*Cdkn1c, Ccdc85a, Myo10, Efnb1, Clic5, Cdh23*) in BAT-ECs (Supplementary Fig. 1D–S). We also assessed the 15 most significantly enriched genes in WATg-ECs compared to BAT-ECs. We explored their expression in the BAT dataset [23] and found that several genes were enriched in the EC subsets, particularly in lymphatic endothelial cells (LECs) (*Col23a1*, *Scn5a*, *Sned1*, *Prox1*, *Pcsk6*, *Tll1*, *Loxl2*, *Pard6g*), while for the remaining ones no EC-enrichment was detected or the genes (*Leprel1*, *Efemp1*) were not found in the dataset (Supplementary Fig. 2A-M).

We next examined whether the BAT-EC and WAT-EC enriched genes identified in our murine dataset showed similar expression patterns in human adipose tissue by reanalyzing published single-cell and single-nucleus RNA-seq datasets [24,25] . WAT-EC enriched genes were highly expressed in human visceral adipose tissue (Supplementary Fig. 2N-U) and were also detectable in the endothelial subset of human subcutaneous BAT (Supplementary Fig. 3A–H) [25]. In contrast, BAT-EC enriched genes were predominantly found in ECs of human subcutaneous BAT (Supplementary Fig. 3I-P) and were only minimally expressed in ECs of human subcutaneous WAT (Supplementary Fig. 4A–G) [25]. This cross-species comparison suggests that the transcriptional differences we identified between murine BAT-ECs and WAT-ECs reflect, at least in part, conserved patterns in human adipose tissue. Specifically, genes enriched in murine WAT-ECs appear to represent a broader adipose tissue endothelial program that is present in both human WAT and BAT. In contrast, the genes enriched in murine BAT-ECs show a more restricted pattern, being predominantly enriched in human BAT-ECs and largely absent from human WAT-ECs.

Together, our findings indicate that BAT-EC-associated transcriptional features are more depot-restricted and conserved across species, whereas WAT-EC-enriched genes represent a more general endothelial signature shared across adipose depots.

### 3.5 Thermogenic remodeling of BAT is accompanied by adaptive EC responses

BAT plays a central role in thermogenic energy expenditure and is activated by cold exposure and β-adrenergic stimulation. This activation induces profound metabolic and structural adaptations within the tissue, including increased perfusion, mitochondrial activity, and thermogenic gene expression. Given these dynamic transitions between activation and involution, it is plausible that ECs within BAT must adapt to the changing metabolic and vascular demands of the tissue. Such adaptation would be expected to alter their transcriptome, potentially influencing the expression of the endothelial markers we identified. We therefore asked whether BAT activity modulates the expression of these markers.

To further prioritize functional candidates out of the 8 enriched BAT-EC genes, we assessed whether they were regulated upon BAT activation by chronic cold exposure (7 days at 5 °C) in the scRNASeq dataset [23] (Supplementary Fig. 4H-M). Indeed, *Rgcc*, *Tcf15*, and *Cdkn1c* were significantly regulated under cold exposure. Therefore, we focused on these genes to determine whether they have been previously linked to endothelial or adipose tissue biology. We also examined their endothelial expression after β-adrenergic stimulation, and long-term cold exposure—the latter characterized by increased angiogenesis [26].

**Figure 3:**
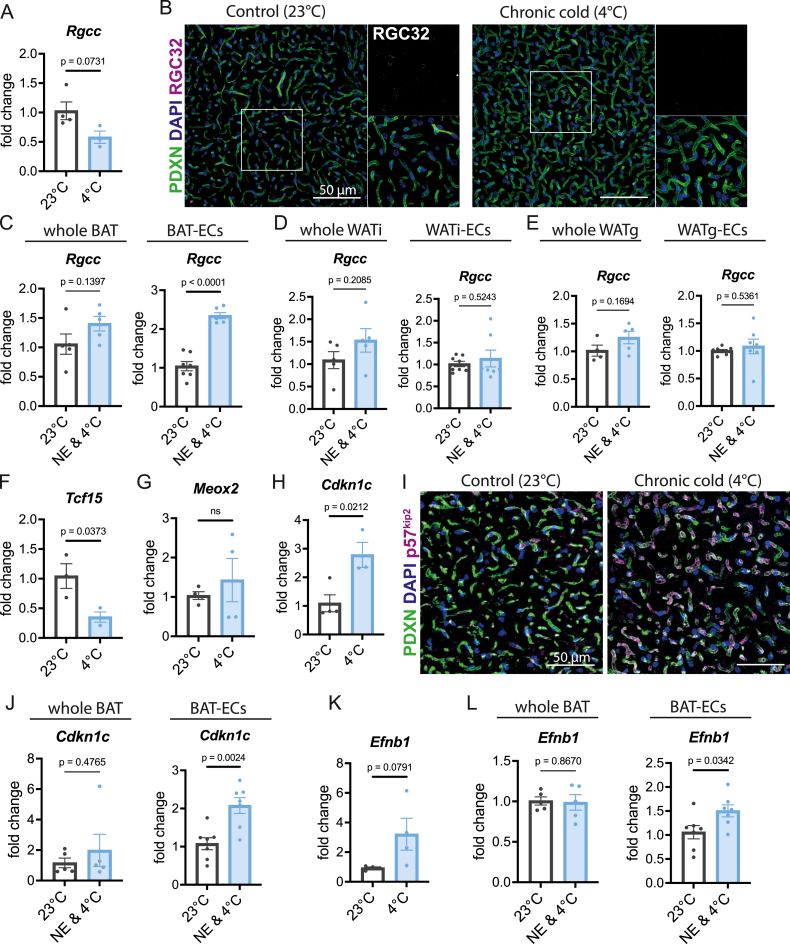
**BAT-EC enriched markers are regulated by β-adrenergic stimulation and/or cold exposure A** mRNA levels of *Rgcc* in BAT-ECs isolated of C57Bl6 mice housed at 23 °C or 4 °C (n ≥ 3 independent samples each condition) detected using qPCR. **B** Confocal images of BAT labelled with a Podocalyxin (PDXN), DAPI and RGC32-specific antibody. Magnified cutouts on the right display decreased RGC32 puncta in BAT of C57Bl6 mice exposed to chronic cold compared to Control. **C**, **D**, **E***Rgcc* transcript levels in whole lysate (left) or purified ECs (right) of BAT (**C**), WATi (**D**) and WATg (**E**) in C57Bl6 mice housed at room temperature in comparison to mice injected with noradrenalin and house at 4 °C for 1 h. **F, G, H** qPCR results of *Tcf15* (**G**), *Meox2* (**H**) and *Cdkn1c* (**I**) from isolated BAT-ECs from Control or mice housed at 4 °C for one week (n ≥ 3 independent samples). **I** Podocalyxin (PDXN), DAPI and p57^kip2^ immunofluorescence labeled BAT of C57Bl6 mice housed at 23 °C or 4 °C, presenting increased and endothelial specific p57^kip2^ fluorescence in the 4 °C condition. **J***Cdkn1c* transcript levels in whole lysate (left) or purified BAT-ECs (right) in C57Bl6 mice housed at room temperature in comparison to mice injected with noradrenalin and house at 4 °C for 1 h (n ≥ 5 independent samples each condition). **K** Transcript levels of *Efnb1* in BAT-ECs of C57Bl6 mice housed at 23 °C or 4 °C (n = 4 independent samples each condition) detected using qPCR. **L***Efnb1* transcript levels in whole lysate (left) or purified BAT-ECs (right) in C57Bl6 mice housed at room temperature in comparison to mice injected with noradrenalin and house at 4 °C for 1 h (n ≥ 5 independent samples each condition). For **A**, **C-E**, **F–H** and **J-L** data represent mean ± s.e.m.; two-tailed unpaired t-test. The numerical data and *P* values are provided in figure. *P* values lower that 0.05 are considered as significant.

Rgcc (Regulator of Cell Cycle; also known as RGC-32) is broadly expressed and has been reported to enhance proliferation by repressing cell-cycle inhibitors, but in some cancer cells it inhibits mitosis and blocks tumor growth [27]. In ECs, RGC-32 has been described as a capillary marker and as a hypoxia-induced regulator that limits proliferation, migration, and angiogenesis [1,28]. Intriguingly, RGC-32 knockout mice display increased browning and energy expenditure, protecting against diet-induced obesity [29]. In our reanalysis, endothelial *Rgcc* expression was significantly reduced under long-term cold exposure (Supplementary Fig. 4H). We validated this finding by measuring *Rgcc* levels in freshly isolated BAT-ECs before and after long-term cold exposure (Figure 3A). In addition, immunostaining of BAT sections using an anti–RGC-32 antibody revealed nuclear RGC-32–positive puncta, which were markedly diminished in BAT from cold-exposed mice (Figure 3B). However, β-adrenergic stimulation followed by 1 h of cold exposure resulted in a significant increase in *Rgcc* expression in BAT-ECs (Figure 3C), while on whole BAT level there are only moderate changes. Further this effect is specific to BAT, as WATi-ECs or WATg-ECs do not show this response (Figure 3D–E).

Overall, these results identify RGC-32 as a dynamically regulated endothelial marker in BAT, whose expression is acutely induced by adrenergic stimulation but diminished during prolonged cold exposure and metabolic stress.

TCF15 (Transcription Factor 15) regulates fatty acid uptake in cardiac ECs, where it heterodimerizes with MEOX2 to control cardiomyocyte metabolism [30]. Coppiello *et al.* showed that TCF15 and MEOX2 are highly expressed in ECs of tissues with strong reliance on fatty acid metabolism, including BAT, WAT, and heart [30]. Consistent with this, our bulk RNA-seq data detected abundant *Meox2* in both BAT- and WATg-ECs, although not differentially regulated. Reanalysis of single-cell RNA-seq revealed significant downregulation of *Tcf15,* but not *Meox2* upon cold exposure, which we confirmed in isolated BAT-ECs (Figure 3F–G). β-adrenergic activation reduced *Tcf15* and *Meox2* levels in whole lysates of BAT and even more prominently in WATg, but this was not an EC-derived response, as endothelial *Tcf15* and *Meox2* levels were not affected in BAT or WATg (Supplementary Fig. 5A–F). Together, these results suggest that the cold- and β-adrenergic–induced changes in *Tcf15* and *Meox2* expression occur predominantly at the tissue level rather than within the endothelial compartment. They further indicate that *Tcf15* and *Meox2* are not exclusively expressed in ECs of adipose tissues, but are also contributed by non-EC populations.

Cdkn1c (Cyclin-dependent kinase inhibitor 1c; p57^Kip2^) is a well-established negative regulator of cell cycle progression. Previous studies demonstrated its high abundance in BAT compared to WATi and WATg [31]. In our analysis, *Cdkn1c* was enriched in BAT-ECs and further upregulated after we exposed mice one week to 4 °C, prompting further validation (Figure 3H). Immunostaining of BAT from mice housed at room temperature or after one week of cold exposure revealed strong p57^kip2^ signal in cold-exposed BAT, colocalizing with the endothelial marker Podocalyxin (PDXN) (Figure 3I). Consistent with this, immunoblotting of whole AT lysates showed increased p57^kip2^ levels in BAT upon cold exposure, whereas levels in WATi and WATg remained largely unchanged (Supplementary Fig. 6A–F). Finally, we examined whether *Cdkn1c* responds to β-adrenergic stimulation. *Cdkn1c* levels were unchanged at the whole-tissue level in BAT, WATi and WATg; however, *Cdkn1c* was significantly upregulated in BAT-ECs upon NE-stimulation (Figure 3J; Supplementary Fig. 5G–H). Collectively, these findings suggest that p57^kip2^ functions as a cold- and β-adrenergically responsive regulator within the endothelial compartment of BAT, potentially contributing to the control of endothelial proliferation and remodeling during thermogenic adaptation.

Although *Efnb1* was not significantly regulated by cold exposure in published snRNA-seq data [23], we explored it further as Ephrins have been shown to be important in ECs. Ephrin-B1 (*Efnb1*), a ligand of EphB receptors, regulates cell–cell communication and angiogenesis. Low *Efnb1* levels in murine AT have been linked to obesity and inflammation [32]. We found that EFNB1 protein abundance significantly increased in BAT after one week of cold exposure, consistent with elevated *Efnb1* expression in isolated BAT-ECs (Figure 3K; Supplementary Fig. 6A and B). Conversely, EFNB1 decreased in WATi and WATg under the same conditions (Supplementary Fig. 6C–F). Under acute β-adrenergic stimulation *Efnb1* levels are unchanged in BAT, WATi and WATg (Figure 3L; Supplementary Fig. 5I–J). However, it is significantly increased in BAT-ECs in an acute response to Noradrenalin and cold exposure (Figure 3L). Immunostaining further revealed that EFNB1 expression within BAT vasculature is heterogeneous, with only a subset of endothelial cells displaying high EFNB1 abundance (Supplementary Fig. 5K). This opposing depot-specific regulation, combined with its patchy endothelial expression pattern, suggests that EFNB1 may mark specialized endothelial subpopulations and may participate in depot-specific vascular adaptations to cold.

In summary, our approach identified several genes enriched in BAT-ECs and WATg-ECs relative to their respective counterpart, with several of these signatures also conserved in human adipose tissue. Among the BAT-EC-enriched genes, RGC-32, p57^kip2^, and EFNB1 were directly induced by β-adrenergic stimulation, underscoring specialized endothelial functions that support thermogenic adipose tissue. In contrast, although TCF15 and MEOX2 were highly expressed in BAT-ECs, their expression remained unchanged within the endothelial compartment upon stimulation, while being strongly suppressed in whole adipose tissue. This indicates that TCF15 and MEOX2 do not contribute to thermogenic adaptations specifically at the endothelial level.

### 3.6 Transcriptome of freshly isolated versus cultured ECs

**Figure 4:**
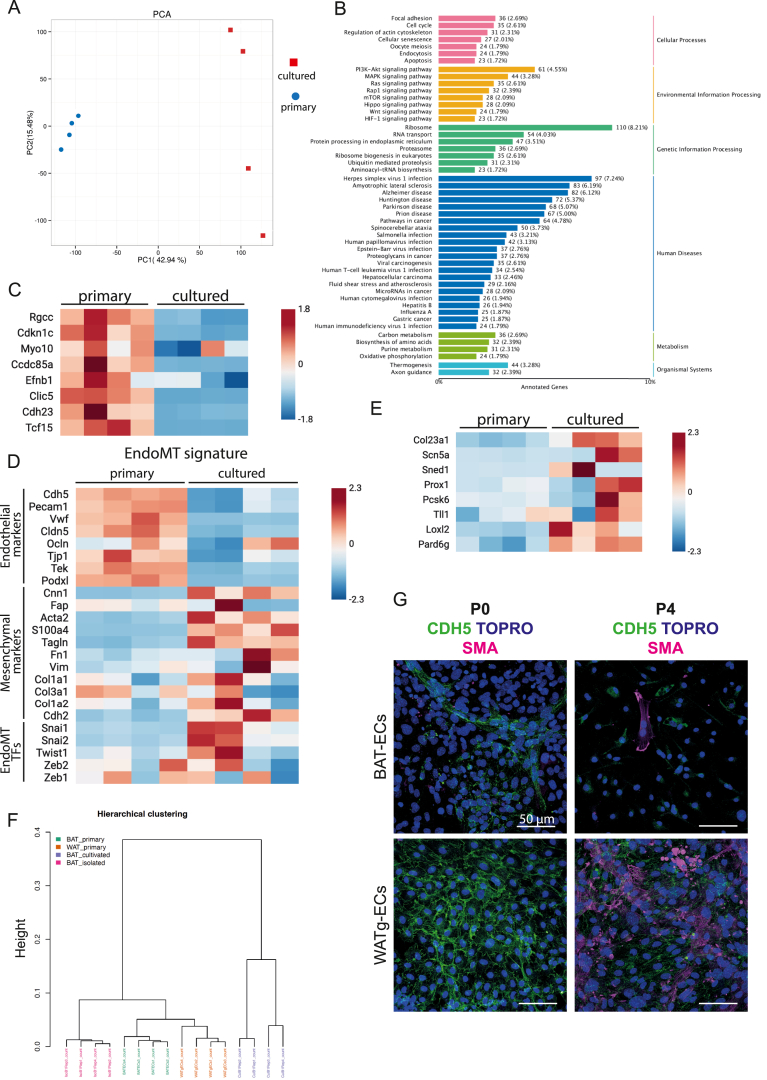
**Cultured BAT-ECs lose their niche-specific transcriptome and gain WAT-EC characteristics A** Principal Component Analysis (PCA) of 4 replicates of cultured and primary endothelial cells isolated from BAT. This PCA shows that the transcriptome of primary BAT-ECs clusters closely together, while cultured BAT-ECs differ clearly in PC2 and separate from primary BAT-ECs on PC1. **B** KEGG pathway analysis using all significantly upregulated genes in BAT-ECs cultured for one passage compared to primary BAT-ECs. **C** Heatmap comparing the transcript levels of defined BAT-EC markers from primary BAT-ECs versus cultured BAT-ECs. **D** Heatmap of genes involved in endothelial-to-mesenchymal transition (EndoMT) revealing that endothelial markers decrease under culture conditions, while mesenchymal marker and EndoMT transcription factors increase. This suggests that BAT-ECs undergo EndoMT *in vitro*. **E** Heatmap of mRNA levels of defined WAT-EC markers in primary BAT-ECs compared to cultured BAT-ECs. This implicates that BAT-ECs transition to a WAT-ECs transcriptome in culture. **F** Hierarchical clustering of transcriptome data from primary BAT-ECs, primary WAT-ECs and cultured BAT-ECs. Indicating that the transcriptome of cultured BAT-ECs is closer to primary WAT-ECs than primary BAT-ECs. **G** Representative confocal images of BAT-ECs (upper panels) and WATg-EC (lower panels) culture undergoing EndoMT after 4 passages. EndoMT is identified by the loss of CDH5 staining and gain of smooth muscle actin (SMA) staining.

The aim of this study was not only to validate our BAT-EC isolation method and endothelial genes particularly abundant in BAT-ECs, but also to analyze potential changes during culture. Thus, we compared freshly isolated BAT-ECs with BAT-ECs cultured over one passage. PCA showed that freshly isolated BAT-ECs clustered tightly along PC1 and PC2, indicating a highly similar transcriptome, whereas cultured BAT-ECs clustered together on PC1 but shifted markedly along PC2, suggesting that culture conditions diversify their transcriptome (Figure 4A). KEGG analysis revealed significant upregulation of pathways related to focal adhesion and cell cycle regulation, which reflects the expected adaptation of ECs to *in vitro* conditions, where they must proliferate, grow, and adhere to culture dishes. Pathways such as PI3K–AKT and mTOR signaling, which support EC proliferation and growth, were also enriched, as well as metabolic pathways associated with nucleotide and amino acid synthesis—critical for cellular mass doubling (Figure 4B, Supplementary Table 1). Interestingly, thermogenesis-related genes were also upregulated, suggesting that cultured cells retain elements associated with BAT function (Figure 4B, Supplementary Table 1).

To explore this further, we examined mRNA levels of BAT-EC-enriched genes newly defined in this study. All were significantly downregulated under culture conditions (Figure 4C). Given that many of these markers suppress cell cycle progression, their downregulation is consistent with the proliferative drive of cells under culture conditions. Moreover, *in vitro* BAT-ECs lack blood flow, their native microenvironment, and surrounding smooth muscle cells, all factors that can trigger an endothelial-to-mesenchymal transition (EndoMT), as previously described [10]. Indeed, transcriptomic analysis revealed an increase in EndoMT-associated gene signatures in cultured BAT-ECs (Figure 4D). Aditionally, cultured BAT-ECs increase WAT-EC enriched genes defined in this study (Figure 4E). Consistent with this notion, Pearson correlation analysis demonstrated that the global transcriptional profile of cultured BAT-ECs is more similar to that of primary WAT-ECs than to primary BAT-ECs (Figure 4F). This shift towards a WAT-EC-like state is further reflected by the downregulation of canonical endothelial marker genes alongside the upregulation of mesenchymal genes and transcription factors known to drive epithelial-to-mesenchymal transition (EMT) (Figure 4E).

To assess whether these transcriptional changes are reflected at the protein level, we analyzed BAT-EC cultures by immunofluorescence staining for the endothelial marker VE-cadherin (CDH5) and the mesenchymal marker α–smooth muscle actin (SMA). Early-passage cultures (passage 0 (p0)) exhibited robust CDH5 staining with little to no detectable SMA, whereas later passages (p4) showed markedly reduced CDH5 signal accompanied by increased SMA expression (Figure 4G).

Given the progressive loss of canonical endothelial markers and BAT-EC-enriched genes in cultured BAT-ECs, we considered whether these transcriptional changes reflect a nonspecific dedifferentiation process commonly observed under *in vitro* conditions, or instead indicate a more directed shift toward an alternative endothelial state. While our data do not allow us to exclude dedifferentiation as a contributing factor, transcriptomic comparisons provide clear evidence that cultured BAT-ECs are transcriptionally more similar to primary WAT-ECs than to primary BAT-ECs.This observation suggests that the transcriptional state adopted by BAT-ECs in culture may represent a less specialized endothelial phenotype that resembles WAT-ECs, rather than a complete loss of endothelial identity. In this context, BAT-ECs appear to exhibit a high degree of specialization *in vivo*, whereas the WAT-EC state may reflect a more basal or permissive endothelial program. Together, these observations raise the possibility that the EndoMT-like signature observed in cultured BAT-ECs reflects an adaptive transcriptional response that parallels aspects of “whitening” at the endothelial level, rather than nonspecific dedifferentiation alone. This interpretation underscores the plasticity of BAT-ECs and suggests that endothelial identity in BAT may be tightly coupled to the specialized metabolic state of the tissue.

## 4 Discussions

Studying the vasculature in adipose tissue faces major hurdles [33]. In particular, the study and identification of angiokines and batokines along with their mechanisms of action is extremely challenging. *In vivo* studies require genetically modified mice and sophisticated and expensive technology [34,35]. Additionally, the results are highly dependent on the endothelial-specific Cre-recombinase. As there is so far no recombinase that targets only the vasculature of AT, the interpretation of the results is extremely challenging and it is hard to exclude the contribution from other organs.

Thus, isolating and culturing ECs from AT enables in-depth investigation of their unique characteristics, signaling pathways, and interactions within the microenvironment of brown, beige and white fat. However, due to the small size and complex structure of adipose depots, isolating pure populations of viable ECs and bringing them into culture presents a distinct set of challenges.

While several published protocols describe EC isolation, none fully met the specific requirements. Many focus on isolating ECs from the lung, heart, or aorta, often involving complex perfusion techniques that are difficult to implement in small adipose depots [20,21,[36], [37], [38]]. Others describe EC isolation from human or mouse tissues but lack a focus on long-term culture conditions, a critical aspect for functional studies [[39], [40], [41]]. Additionally, commercially available primary or immortalized ECs predominantly originate from the mouse lung, human umbilical cord, or skin. These ECs lack the necessary characteristics of AT-derived ECs, making them unsuitable for investigating the signaling mechanisms between ECs and adipocytes.

Our protocol yields approximately 80% pure ECs from both BAT and WAT. Moreover, this study identified endothelial genes enriched in BAT relative to WAT that are dynamically regulated by cold and β-adrenergic stimulation: *Rgcc*, *Cdkn1c*, *Tcf15*, *Meox2* and *Efnb1*.

Rgcc (or RGC-32) is a VEGF and hypoxia-induced protein that inhibits angiogenesis [28] . Interestingly, the RGC-32 KO mouse displays increased browning and energy expenditure, protecting the mouse from obesity [29]. The authors did not find a major functional change in RGC-32 knockdown adipocytes, suggesting that other cells in AT are affected by the loss of RGC-32. Our reanalysis of single-cell RNASeq data showed that endothelial *Rgcc* levels significantly drop in mice exposed to chronic cold. However, β-adrenergic stimulation leads to increased *Rgcc* expression in BAT, while this is not detected in whole tissue lysates or other adipose depots. This transient and depot-specific upregulation of *Rgcc* may reflect an early response to β-adrenergic activation and potentially to acute tissue hypoxia, a phenomenon previously associated with the initial stages of cold exposure [26]. In contrast, prolonged cold exposure necessitates increased vascularization and angiogenesis. Because RGC-32 is known to restrict angiogenic processes [28], its sustained suppression during long-term cold exposure may help facilitate the angiogenic remodeling required for BAT adaptation. Increased vascularization supports BAT [42] and together this could explain the maintained browning and resistance to HFD-induced obesity in the RGC-32 KO mice.

Our data indicate that although TCF15 and MEOX2 are abundantly present in adipose tissue ECs, cold- and β-adrenergic–induced changes in their expression occur primarily at the tissue level and are not driven by ECs. This is notable given that the TCF15–MEOX2 heterodimer has previously been linked to endothelial fatty acid uptake in metabolically active organs [30] . Together, our findings suggest that regulation of the TCF15/MEOX2 axis in adipose tissues is largely non–endothelial-intrinsic and may reflect broader metabolic adaptations to thermogenic activation.

P57^kip2^ (Cdkn1c) is known to inhibit cell cycle progression. Mutations in this gene are common in the growth restriction disorder Silver Russell Syndrome, IMAGe Syndrome and Beckwith Wiedemann Syndrome [43]. Besides the strong growth retardation, these patients show significantly reduced adiposity. A mouse model of Cdkn1c overexpression, mimicking the described disorders, has substantially more BAT, while the Cdkn1c KO mouse fails to develop BAT [43]. In particular, the Cdkn1c KO mouse fails to accumulate PRDM16 and thus to develop brown/beige adipocytes. In human adipose-derived stem cells p57^Kip2^ was shown to inhibit key cell cycle molecules, thereby inducing quiescence or even senescence [44]. Another related cell cycle inhibitor, p27^Kip1^, which shares the same degradation pathway, has been shown to restrict proliferation in mature brown adipocytes and, consequently, affect thermogenic adaptation in BAT. This study also reported that p57^Kip2^ is exclusively expressed in BAT compared to gonadal white adipose tissue (WATg) and inguinal WAT (WATi) [31]. Despite this, only a single study has suggested that Cdkn1c may be enriched in endothelial cells (ECs). Specifically, p57^Kip2^ was described as a downstream target of KLF2 and KLF4, and its expression was upregulated by laminar flow, though this regulation was specific to lymphatic ECs [45]. Notably, blood ECs did not show a similar flow-induced increase in *Cdkn1c* expression [45].

Building on these established roles of *Cdkn1c* in adipose tissue development and thermogenic function, our data uncover an additional, previously underappreciated endothelial component of p57^Kip2^ regulation in BAT. We find that *Cdkn1c* is enriched in BAT-ECs and further induced by cold exposure, with p57^Kip2^ localizing specifically to the BAT vasculature. Moreover, while whole-tissue *Cdkn1c* levels were largely insensitive to β-adrenergic stimulation, endothelial *Cdkn1c* was selectively upregulated in BAT following norepinephrine exposure, indicating cell type–specific regulation. Together, these findings extend previous observations of *Cdkn1c* expression beyond adipocytes and identify p57^Kip2^ as a cold- and β-adrenergically responsive factor within the BAT-EC compartment. Given its established role as a cell cycle inhibitor, endothelial p57^Kip2^ may contribute to the fine-tuning of endothelial proliferation and vascular remodeling during thermogenic adaptation.

Ephrin-B1 (encoded by Efnb1) is a transmembrane ligand within the B-class ephrin family, engaging in bidirectional signaling with EphB receptors to orchestrate cell–cell communication. In ECs, ephrin–Eph interactions are pivotal in vascular development and angiogenesis: for instance, ephrin-B1 is expressed in arterial and venous endothelial cells and contributes to capillary sprouting and vessel patterning during vascular morphogenesis [[46], [47], [48]]. Specifically, human coronary artery ECs express ephrin-B1 protein, and its expression can be induced by inflammatory stimuli [32]. Beyond vascular biology, ephrin-B1 was also detected in AT. Expression of ephrin-B1 is notably reduced in adipose tissue—especially in mature white adipocytes—of obese mice, and its downregulation exacerbates adipose inflammation. Overexpressing ephrin-B1 in adipocytes, on the other hand, suppresses inflammatory responses, underscoring a protective role in AT homeostasis [49]. In this context, our findings reveal a previously unrecognized, depot- and cell type–specific regulation of ephrin-B1 in adipose tissue vasculature during thermogenic activation. We show that EFNB1 abundance is selectively increased in BAT following cold exposure and acutely induced in BAT-ECs in response to noradrenergic stimulation, whereas EFNB1 levels are reduced in WAT depots under the same conditions. Notably, endothelial EFNB1 expression in BAT is heterogeneous, with high expression confined to a subset of ECs, suggesting functional specialization within the BAT vasculature. One possible explanation is that in BAT, endothelial EFNB1 upregulation supports angiogenesis and vascular remodeling in response to thermogenic activation, thereby facilitating the increased nutrient and oxygen demands in cold-activated BAT. In WAT, however, downregulation of EFNB1 is likely not mediated by ECs, but other cells, e.g. mature adipocytes [49]. This opposing regulation highlights the depot-specific signaling and suggests that Ephrin–Eph signaling contributes not only to vascular plasticity but also to the functional divergence between BAT and WAT vasculature.

We developed our protocol not only to isolate and enrich BAT-ECs, but also to culture and expand them, which enabled us to examine how BAT-EC identity is maintained or altered under *in vitro* conditions. Cultured BAT-ECs exhibited a progressive reduction in the expression of BAT-EC-enriched genes, many of which are associated with endothelial quiescence and inhibition of cell cycle progression. At the adult stage at which BAT-ECs are isolated, endothelial cells are largely non-proliferative, consistent with their highly specialized *in vivo* function. Upon transfer to culture, however, BAT-ECs are forced to re-enter the cell cycle and undergo a rapid angiogenic switch, a process that is well known to be accompanied by substantial transcriptional remodeling [10] . In this context, the emergence of EndoMT-associated transcriptional signatures in cultured BAT-ECs can be interpreted as an adaptive response to the *in vitro* environment. EndoMT has frequently been reported as a consequence of endothelial cell culture and is thought to facilitate survival, proliferation, and phenotypic flexibility under non-physiological conditions [10,50,51] . Rather than representing a purely artifactual loss of identity, this remodeling may therefore reflect a necessary transition that enables BAT-ECs to exit their highly specialized *in vivo* state and acquire a proliferative phenotype compatible with culture expansion.

At the same time, the transcriptional changes observed in cultured BAT-ECs are not random. Comparative transcriptomic analyses revealed that cultured BAT-ECs become more similar to primary WAT-ECs than to primary BAT-ECs, characterized by loss of BAT-EC-enriched genes and acquisition of a WAT-EC-like transcriptional profile. This convergence suggests that BAT-EC identity is strongly dependent on continuous tissue-specific cues, including thermogenic stimuli and the unique metabolic microenvironment of BAT. In their absence, BAT-ECs appear to revert toward a less specialized endothelial state resembling that of WAT-ECs, which may represent a more basal or permissive endothelial program.

Importantly, such a shift may also be relevant *in vivo*. EndoMT-associated signatures and reduced endothelial specialization have been reported in metabolically challenged tissues, including during aging and obesity [7,52,53] . We therefore propose that the transcriptional state adopted by cultured BAT-ECs may mirror early events that occur during BAT involution in these conditions. In this scenario, BAT-ECs progressively lose their specialized, BAT-supportive functions and transition toward a WAT-EC-like state, potentially contributing to impaired vascular support, reduced thermogenic capacity, and ultimately tissue whitening. These observations raise the possibility that endothelial remodeling is not merely a consequence, but may actively participate in the process of BAT dysfunction.

Finally, while speculative, the pronounced plasticity observed in cultured BAT-ECs underscores the dynamic nature of endothelial identity in BAT. The ability of BAT-ECs to undergo extensive transcriptional remodeling highlights their strong dependence on environmental signals and supports the concept that endothelial state transitions may play a previously underappreciated role in BAT maintenance and decline.

In summary, our work establishes a protocol for the isolation and culture of ECs from brown and white adipose depots, enabling functional and molecular studies of their unique properties. Using this approach, we identified several BAT-enriched endothelial markers with roles in angiogenesis, endothelial cell cycle regulation, and tissue remodeling, and uncovered depot-specific regulation of Cdkn1c and Efnb1 under cold exposure. These findings underscore the high degree of endothelial specialization in adipose tissue and reveal that BAT-ECs, while quiescent *in vivo*, undergo dedifferentiation and adopt a WAT-like transcriptome when forced into proliferation in culture. Together, this study highlights both the potential and the limitations of cultured adipose-derived ECs, offering a valuable platform to dissect depot-specific endothelial signaling while emphasizing the need to carefully interpret results in the context of the *in vivo* microenvironment and passage number.

## CRediT authorship contribution statement

**Tabea Elschner:** Conceptualization, Data curation, Formal analysis, Investigation, Methodology, Validation, Visualization, Writing – original draft. **Stephan Grein:** Data curation, Formal analysis, Resources, Software, Writing – review & editing. **Jana Sander:** Data curation, Formal analysis, Methodology, Writing – original draft. **Staffan Hildebrand:** Data curation, Writing – review & editing. **Lara Heubach:** Data curation, Formal analysis, Methodology. **Nina Pannwitz:** Data curation, Formal analysis, Methodology. **Maria Mircea:** Data curation, Formal analysis, Resources, Software. **Elba Raimundez:** Data curation, Formal analysis, Resources, Software, Writing – review & editing. **Vasiliki Karagiannakou:** Formal analysis, Resources, Writing – review & editing. **Anastasia Georgiadi:** Formal analysis, Resources, Writing – review & editing. **Joerg Heeren:** Writing – review & editing. **Jan Hasenauer:** Funding acquisition, Supervision, Writing – review & editing. **Alexander Pfeifer:** Conceptualization, Funding acquisition, Supervision, Writing – review & editing. **Kerstin Wilhelm-Jüngling:** Conceptualization, Data curation, Formal analysis, Funding acquisition, Investigation, Methodology, Project administration, Resources, Software, Supervision, Validation, Visualization, Writing – original draft, Writing – review & editing.

## Declaration of generative AI and AI-assisted technologies in the manuscript preparation process

During the preparation of this work the author(s) used ChatGPT (*OpenAI*) in order to improve proofreading, readability, and language. After using this tool, the author(s) reviewed and edited the content as needed and take(s) full responsibility for the content of the published article.

## Funding

Work here was funded by the 10.13039/501100001659Deutsche Forschungsgemeinschaft (10.13039/501100001659DFG, 10.13039/501100001659German Research Foundation) – TRR333/1–450149205 to T.E., S.G., S.H., L.H., N.P., M.M., E.R., V.K., A.G., J.H., J.H., A.P., K.W.J.

## Declaration of competing interest

The authors declare that they have no known competing financial interests or personal relationships that could have appeared to influence the work reported in this paper.

## Data Availability

Data will be made available on request.

## Appendix A Supplementary data

Figure S1 **A** Brightfield image of a pure BAT-EC culture representing a single-layer of cobblestone-shaped endothelial cells. Arrowheads point to regions with cobblestone endothelial cell shape. **B** Representative brightfield image of a fibroblast contaminated BAT-EC culture. Arrowheads depict multi-layered cells with a spiky cell shape, representing fibroblasts. **C** Relative transcript levels of *Pecam1*, *Cdh5*, *Erg* and *Vwf* at four passages of neonatal BAT-ECs (left panel) and WAT-ECs (right panel) and in muMECs, displaying that BAT-ECs and WAT-ECs do not lose endothelial marker expression during the four passages and endothelial transcript levels are higher than in muMECs. **D** Uniform Manifold Approximation and Projection (UMAP) displaying the different cell types in BAT. All further violine blots display expression levels of the chosen genes within single cells in the depicted cell types: **E***Gpd1*, **F***Pth1r*, **G***Rgcc*, **H***Thrsp*, **I***Cdkn1c*, **J***Fasn*, **K***Myo10*, **L***Chchd10*, **M***Ccdc85a*, **N***Efnb1*, **O***Tbx5*, **P***Ucp1*, **Q***Clic5*, **R***Cdh23*, **S***Tcf15* Figure S1

Figure S2 All violine blots display expression levels of the chosen gene within single cells in the depicted cell types: , **A***Cfh*, **B***Col23a1*, **C***Scn5a*, **D***Prelp*, **E***Sned1*, **F***Smoc1*, **G***Lrrn4cl*, **H***Prox1*, **I***Pcsk6*, **J***Tll1*, **K***Loxl2*, **L***Igf1*, **M***Pard6g*, Violin plots derived from the publicly available single-cell RNA-sequencing dataset published by Emont et al. [24] showing the expression of WAT-EC-enriched marker genes defined in this study in ECs from human visceral adipose tissue. These plots illustrate the distribution and relative expression levels of the identified WAT-EC markers across cell types of human adipose tissue, demonstrating their conservation and expression in human visceral fat ECs, **N***COL23A1*, **O***SCN5A*, **P***SNED1*, **Q***PROX1*, **R***PCSK6*, **S***TLL1*, **T***LOXL2*, **U***PARD6G*. Figure S2

Figure S3 Violine blots displaying expression levels of our suggested murine WAT-EC enriched markers in human subcutaneous BAT using the dataset of Sun et al. [25]:**A***COL23A1*, **B***SCN5A*, **C***SNED1*, **D***PROX1*, **E***PCSK6*, **F***TLL1*, **G***LOXL2*, **H***PARD6G*, Violine blots displaying expression levels of our suggested murine BAT-EC enriched markers in human su bcutaneous BAT using the dataset of Sun et al. [25]:, **I***RGCC*, **J***CDKN1C*, **K***MYO10*, **L***CCDC85A*, **M***EFNB1*, **N***CLIC5*, **O***CDH23*, **P***TCF15*. Figure S3

Figure S4 Violine blots showing expression levels of our suggested BAT-EC enriched markers in human subcutaneous WAT using the dataset of Sun et al. [25]: **A***RGCC*, **B***CDKN1C*, **C***MYO10*, **D***EFNB1*, **E***CLIC5*, **F***CDH23*, **G***TCF1*5. All violine plots are based on data from Shamsi *et al.* [23] and display transcript levels measured for the respective genes in single cells isolated from mice house at room temperature (RT; left panel) and housed at 5 °C for 7 days (cold7; right panel):, **H***Rgcc*, **I***Cdkn1c*, **J***Efnb1*, **K***Tcf15*, **L***Meox2**,***M** Table summarizing log2 fold-change and adjusted p-value of respective genes within the endothelial cell cluster comparing ECs isolated from mice housed at RT versus housed at cold7. Only significantly changed genes are represented. Figure S4

Figure S5 **A**, **B**, **C***Tcf15* transcript levels in whole lysate (left) or purified ECs (right) of BAT (**A**), WATi (**B**) and WATg (**C**) in C57Bl6 mice housed at room temperature in comparison to mice injected with noradrenalin and house at 4 °C for 1 h (n ≥ 4 independent samples each condition), **D**, **E**, **F***Meox2* transcript levels in whole lysate (left) or purified ECs (right) of BAT (**D**), WATi (**E**) and WATg (**F**) in C57Bl6 mice housed at room temperature in comparison to mice injected with noradrenalin and house at 4 °C for 1 h (n ≥ 4 independent samples each condition), **G, H***Cdkn1c* transcript levels in whole lysate (left) or purified ECs (right) of WATi (**G**) and WATg (**H**) in C57Bl6 mice housed at room temperature in comparison to mice injected with noradrenalin and house at 4 °C for 1 h (n ≥ 4 independent samples each condition), **I, J***Efnb1* transcript levels in whole lysate (left) or purified ECs (right) of WATi (**I**) and WATg (**J**) in C57Bl6 mice housed at room temperature in comparison to mice injected with noradrenalin and house at 4 °C for 1 h (n ≥ 4 independent samples each condition), **K** Podocalyxin (PDXN), DAPI and EFNB1 immunofluorescence labeled BAT of C57Bl6 mice housed at 23 °C or 4 °C, presenting patchy endothelial specific EFNB1 fluorescence in the Control and 4 °C condition. For **A**-**J** data represent mean ± s.e.m.; two-tailed unpaired t-test. The numerical data and *P* values are provided in figure. *P* values lower that 0.05 are considered as significant. Figure S5

Figure S6 **A**, **C, E** Immunoblots representing RGC-32, p57^kip2^ and EFNB1 protein levels at 23 °C and 4 °C in BAT (**A**), WATi (**C**) and WATg (**E**). Further the blot displays CD31 to assess endothelial content, UCP1 to validate the response to 4 °C and Calnexin (CAXN) as housekeeping protein. **B**, **D**, **F** Quantification of protein levels of UCP1, CD31, RGC-32, p57^kip2^ and EFNB1 in BAT (**B**), WATi (**D**) and WATg (**F**) at 23 °C (n = 5 independent samples) and 4 °C (n = 5 independent samples). For **B**, **D** and **F** data represent mean ± s.e.m.; two-tailed unpaired t-test. The numerical data and *P* values are provided in figure. *P* values lower that 0.05 are considered as significant. Figure S6

Supplementary Table 1 Detailed overview of KEGG pathway analysis of Figure 2D-E and 4B including KEGG pathway description and associated gene names. Supplementary Table 1
