# Supplementary figures and images for: From brown to white: Brown adipose tissue endothelial cells whiten in culture conditions
Source: Mol Metab. 2026 Mar 12;107:102349. doi: 10.1016/j.molmet.2026.102349 (PMC13049445; doi:10.1016/j.molmet.2026.102349)

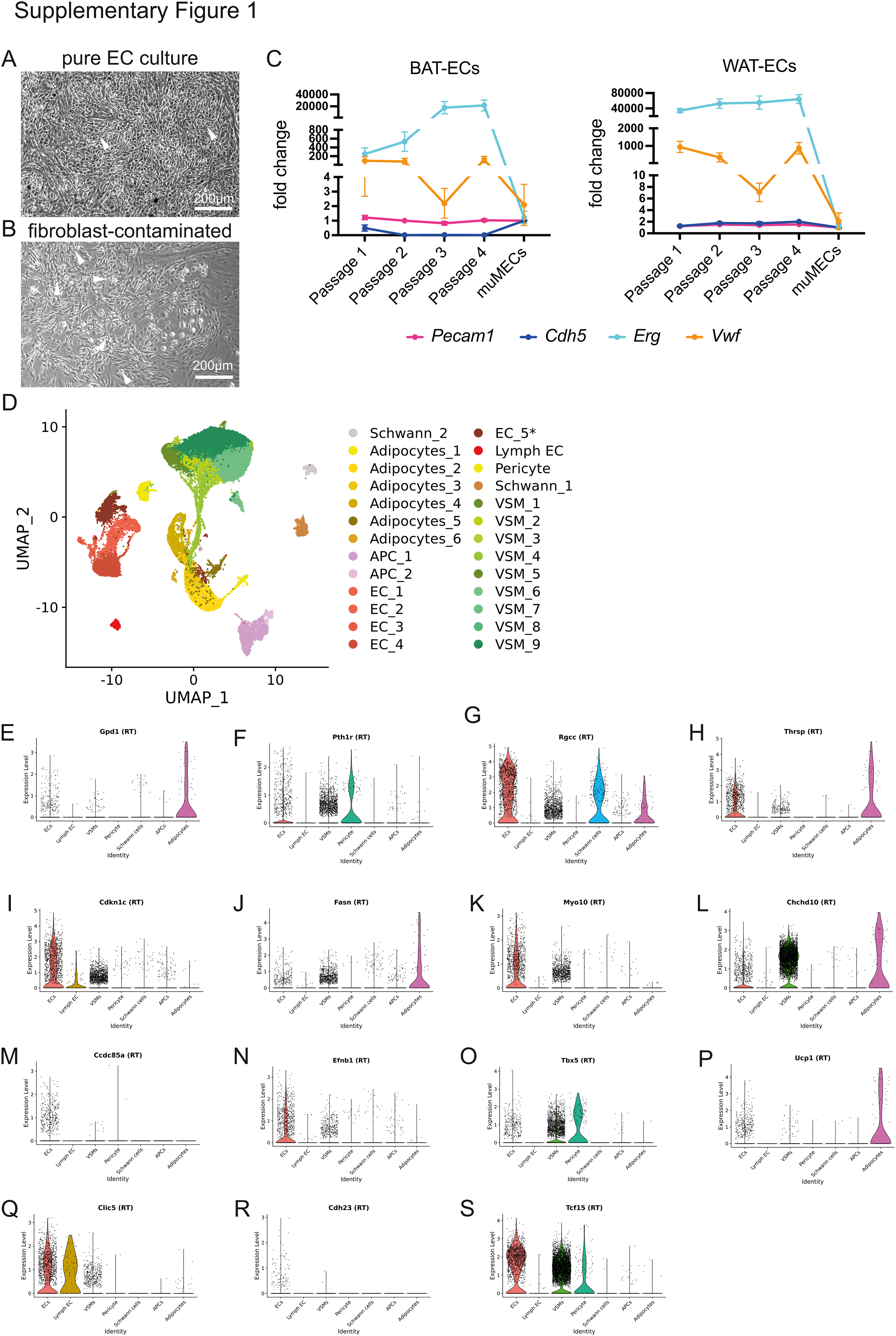

Supplement: Figure S1 — A Brightfield image of a pure BAT-EC culture representing a single-layer of cobblestone-shaped endothelial cells. Arrowheads point to regions with cobblestone endothelial cell shape. B Representative brightfield image of a fibroblast contaminated BAT-EC culture. Arrowheads depict multi-layered cells with a spiky cell shape, representing fibroblasts. C Relative transcript levels of Pecam1, Cdh5, Erg and Vwf at four passages of neonatal BAT-ECs (left panel) and WAT-ECs (right panel) and in muMECs, displaying that BAT-ECs and WAT-ECs do not lose endothelial marker expression during the four passages and endothelial transcript levels are higher than in muMECs. D Uniform Manifold Approximation and Projection (UMAP) displaying the different cell types in BAT. All further violine blots display expression levels of the chosen genes within single cells in the depicted cell types: EGpd1, FPth1r, GRgcc, HThrsp, ICdkn1c, JFasn, KMyo10, LChchd10, MCcdc85a, NEfnb1, OTbx5, PUcp1, QClic5, RCdh23, STcf15 [file figs1.jpg]

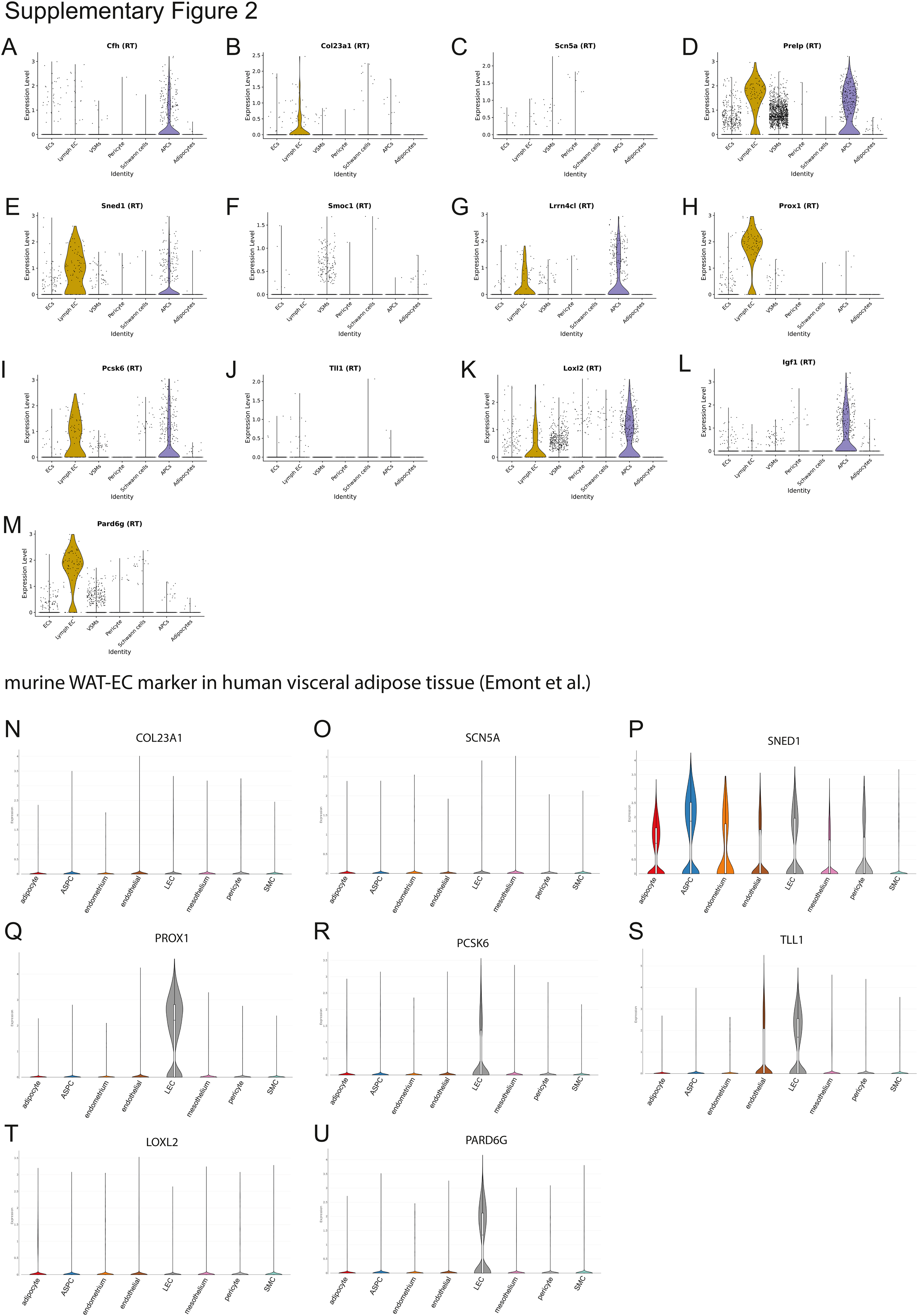

Supplement: Figure S2 — All violine blots display expression levels of the chosen gene within single cells in the depicted cell types: , ACfh, BCol23a1, CScn5a, DPrelp, ESned1, FSmoc1, GLrrn4cl, HProx1, IPcsk6, JTll1, KLoxl2, LIgf1, MPard6g, Violin plots derived from the publicly available single-cell RNA-sequencing dataset published by Emont et al. [24] showing the expression of WAT-EC-enriched marker genes defined in this study in ECs from human visceral adipose tissue. These plots illustrate the distribution and relative expression levels of the identified WAT-EC markers across cell types of human adipose tissue, demonstrating their conservation and expression in human visceral fat ECs, NCOL23A1, OSCN5A, PSNED1, QPROX1, RPCSK6, STLL1, TLOXL2, UPARD6G. [file figs2.jpg]

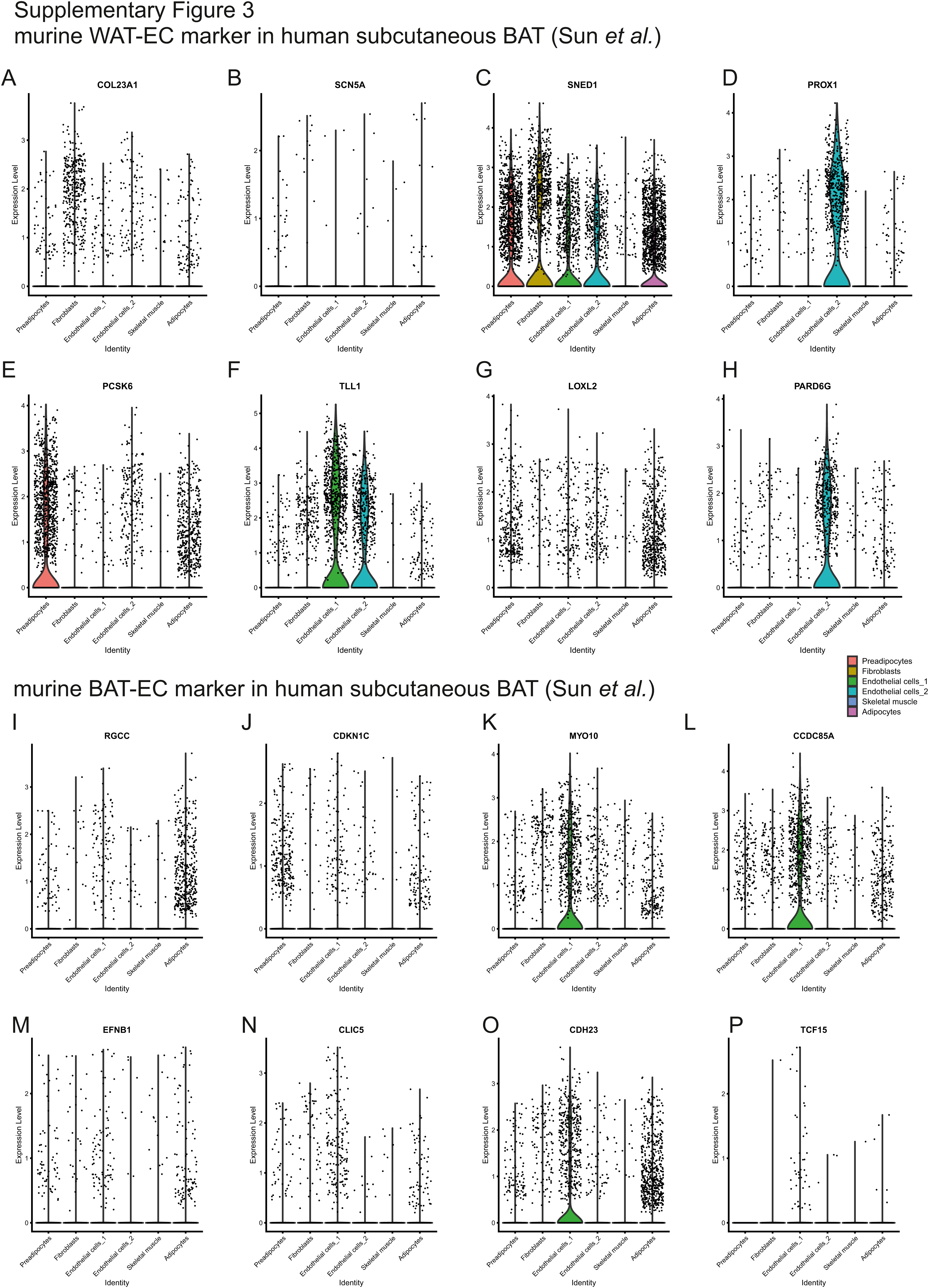

Supplement: Figure S3 — Violine blots displaying expression levels of our suggested murine WAT-EC enriched markers in human subcutaneous BAT using the dataset of Sun et al. [25]:ACOL23A1, BSCN5A, CSNED1, DPROX1, EPCSK6, FTLL1, GLOXL2, HPARD6G, Violine blots displaying expression levels of our suggested murine BAT-EC enriched markers in human su bcutaneous BAT using the dataset of Sun et al. [25]:, IRGCC, JCDKN1C, KMYO10, LCCDC85A, MEFNB1, NCLIC5, OCDH23, PTCF15. [file figs3.jpg]

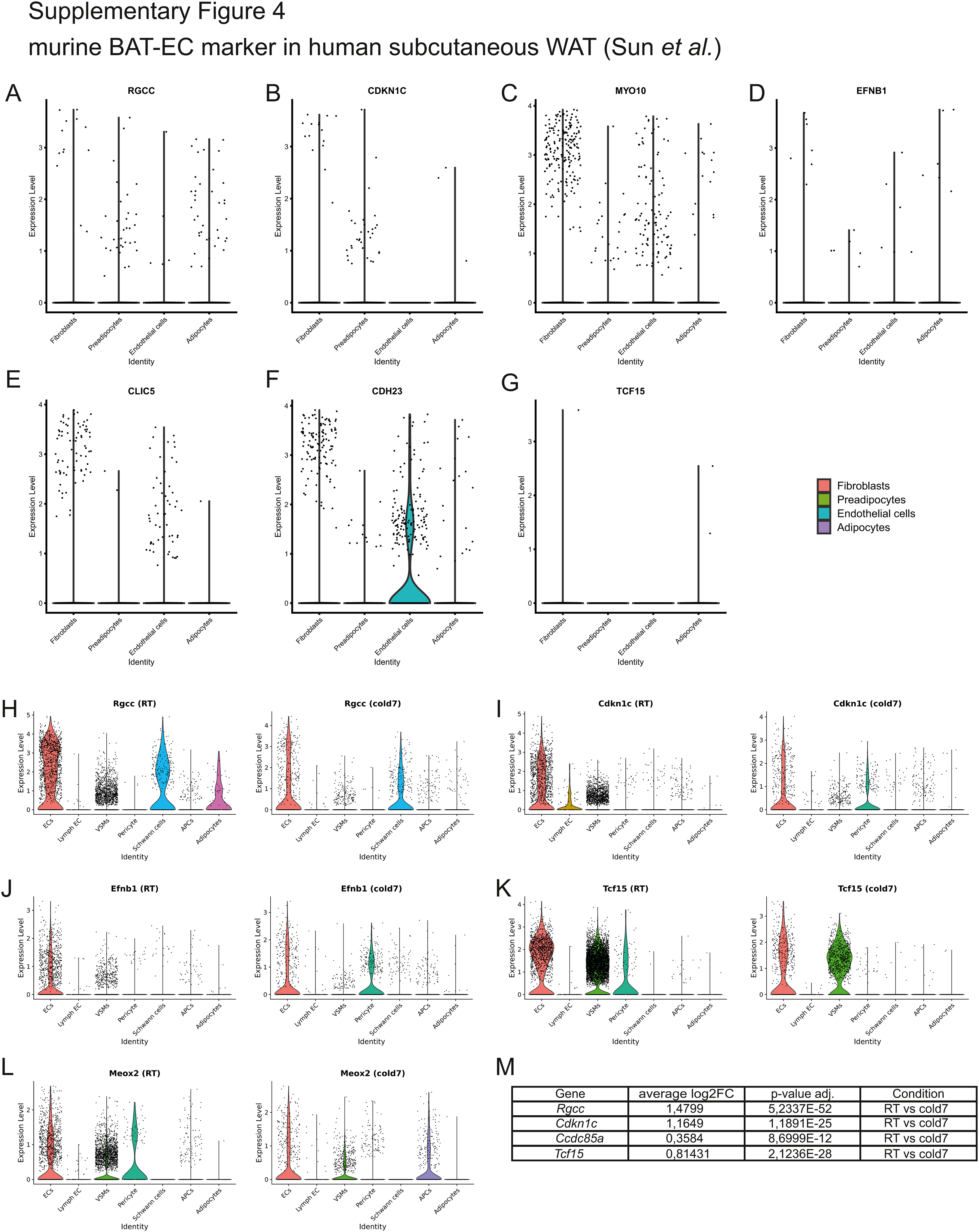

Supplement: Figure S4 — Violine blots showing expression levels of our suggested BAT-EC enriched markers in human subcutaneous WAT using the dataset of Sun et al. [25]: ARGCC, BCDKN1C, CMYO10, DEFNB1, ECLIC5, FCDH23, GTCF15. All violine plots are based on data from Shamsi et al. [23] and display transcript levels measured for the respective genes in single cells isolated from mice house at room temperature (RT; left panel) and housed at 5 °C for 7 days (cold7; right panel):, HRgcc, ICdkn1c, JEfnb1, KTcf15, LMeox2,M Table summarizing log2 fold-change and adjusted p-value of respective genes within the endothelial cell cluster comparing ECs isolated from mice housed at RT versus housed at cold7. Only significantly changed genes are represented. [file figs4.jpg]

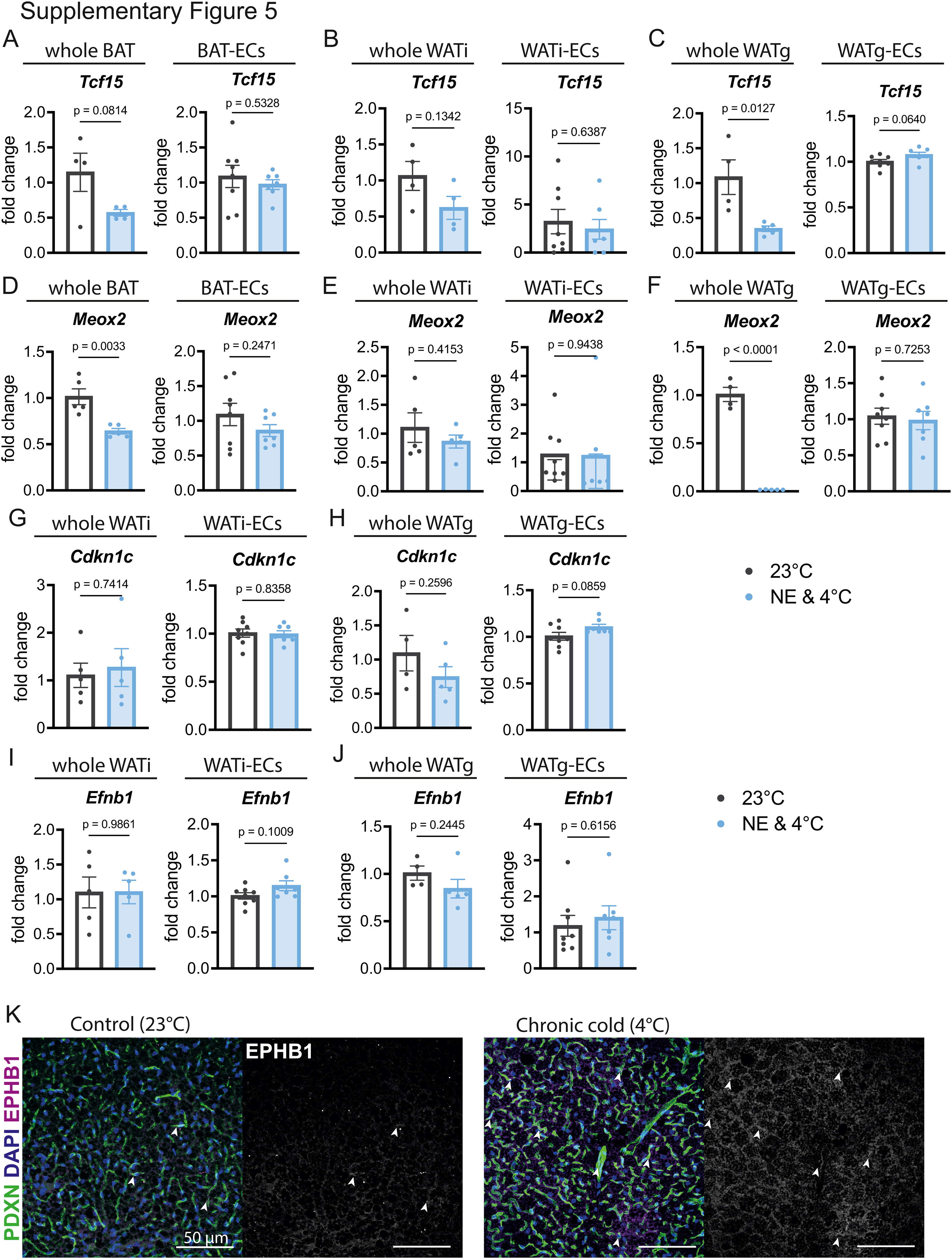

Supplement: Figure S5 — A, B, CTcf15 transcript levels in whole lysate (left) or purified ECs (right) of BAT (A), WATi (B) and WATg (C) in C57Bl6 mice housed at room temperature in comparison to mice injected with noradrenalin and house at 4 °C for 1 h (n ≥ 4 independent samples each condition), D, E, FMeox2 transcript levels in whole lysate (left) or purified ECs (right) of BAT (D), WATi (E) and WATg (F) in C57Bl6 mice housed at room temperature in comparison to mice injected with noradrenalin and house at 4 °C for 1 h (n ≥ 4 independent samples each condition), G, HCdkn1c transcript levels in whole lysate (left) or purified ECs (right) of WATi (G) and WATg (H) in C57Bl6 mice housed at room temperature in comparison to mice injected with noradrenalin and house at 4 °C for 1 h (n ≥ 4 independent samples each condition), I, JEfnb1 transcript levels in whole lysate (left) or purified ECs (right) of WATi (I) and WATg (J) in C57Bl6 mice housed at room temperature in comparison to mice injected with noradrenalin and house at 4 °C for 1 h (n ≥ 4 independent samples each condition), K Podocalyxin (PDXN), DAPI and EFNB1 immunofluorescence labeled BAT of C57Bl6 mice housed at 23 °C or 4 °C, presenting patchy endothelial specific EFNB1 fluorescence in the Control and 4 °C condition. For A-J data represent mean ± s.e.m.; two-tailed unpaired t-test. The numerical data and P values are provided in figure. P values lower that 0.05 are considered as significant. [file figs5.jpg]

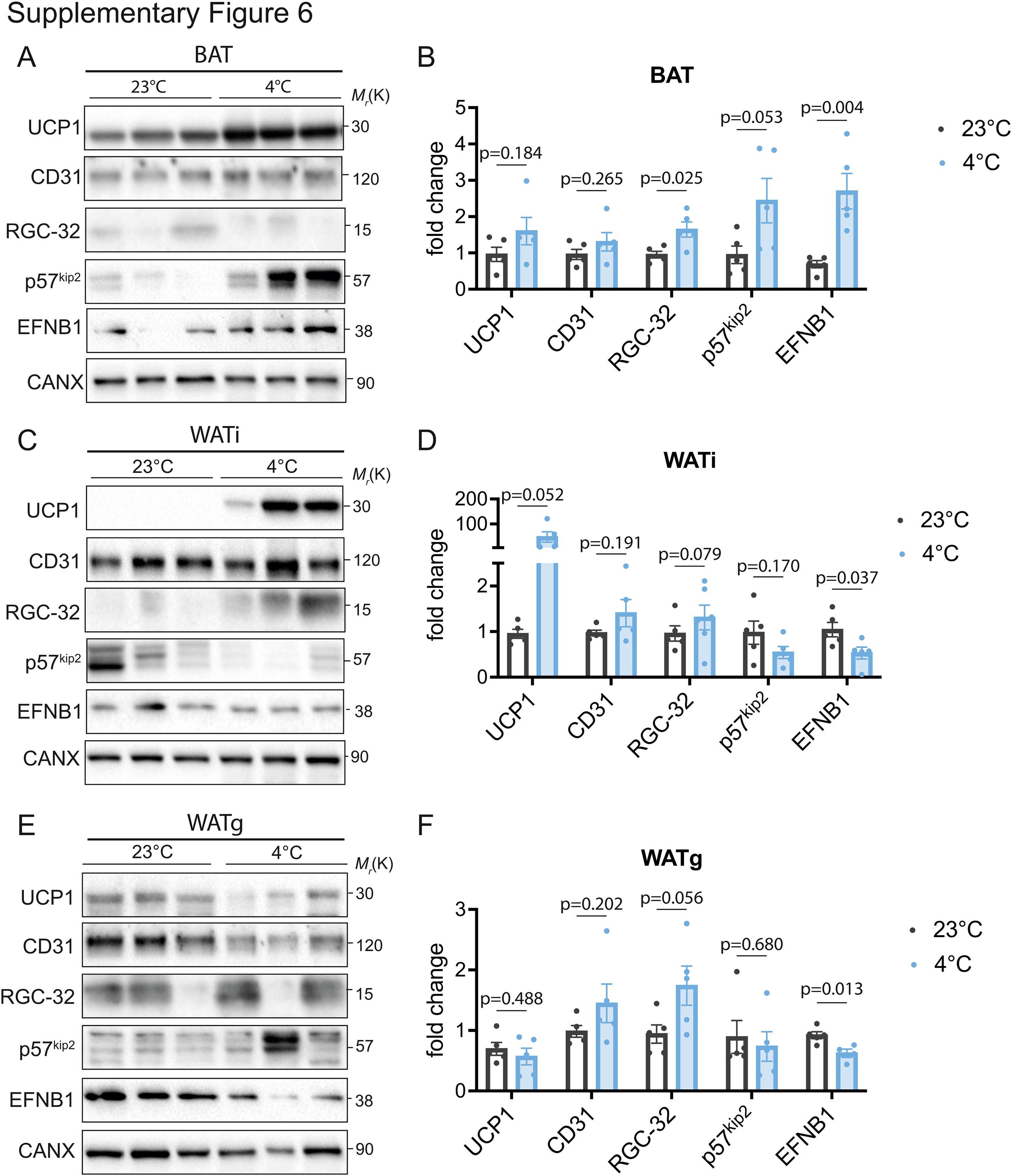

Supplement: Figure S6 — A, C, E Immunoblots representing RGC-32, p57kip2 and EFNB1 protein levels at 23 °C and 4 °C in BAT (A), WATi (C) and WATg (E). Further the blot displays CD31 to assess endothelial content, UCP1 to validate the response to 4 °C and Calnexin (CAXN) as housekeeping protein. B, D, F Quantification of protein levels of UCP1, CD31, RGC-32, p57kip2 and EFNB1 in BAT (B), WATi (D) and WATg (F) at 23 °C (n = 5 independent samples) and 4 °C (n = 5 independent samples). For B, D and F data represent mean ± s.e.m.; two-tailed unpaired t-test. The numerical data and P values are provided in figure. P values lower that 0.05 are considered as significant. [file figs6.jpg]
